# Development of whole-limb skeletal patterning through the coordination of growth and self-organization models

**DOI:** 10.1371/journal.pcbi.1014348

**Published:** 2026-07-07

**Authors:** Soha Ben Tahar, Ester Comellas, Timothy Duerr, Dareen Bakr, James Monaghan, Jose J. Muñoz, Sandra J. Shefelbine

**Affiliations:** 1 Department of Mechanical Engineering, Northeastern University, Boston, Massachusetts, United States of America; 2 Serra Húnter Fellow, Department of Physics, Universitat Politècnica de Catalunya, Barcelona, Spain; 3 International Center for Numerical Methods in Engineering (CIMNE), Barcelona, Spain; 4 Department of Biology, Northeastern University, Boston, Massachusetts, United States of America; 5 Institute for Chemical Imaging of Living Systems, Northeastern University, Boston, Massachusetts, United States of America; 6 Department of Mathematics, Universitat Politècnica de Catalunya, Barcelona, Spain; 7 Department of Bioengineering, Northeastern University, Boston, Massachusetts, United States of America; University of A Coruna: Universidade da Coruna, SPAIN

## Abstract

The vertebrate limb provides an interesting system to study how tissue growth and molecular signaling interact to shape complex skeletal patterns. How these processes are coordinated across space and time is not fully understood. This study introduces a computational tool to examine how growth interacts with positional cues and self-organizing patterning mechanisms to shape skeletal structures in both mice and axolotl limbs. We developed the Growth-Reaction-Diffusion (GRD) framework, a reaction-diffusion system within a growing domain, where reaction represents the regulation of patterning cues and diffusion captures their spatial propagation. The relative contribution of growth, reaction and diffusion is modulated through two non-dimensional parameters, whose spatial variation is informed by positional cues derived from experimental morphogen maps. This formulation normalizes the reaction-diffusion equation relative to growth, enabling investigation of how different spatiotemporal regimes of growth interact with reaction and diffusion to produce whole limb patterning. The GRD framework captures the progressive formation of all limb segments: the humerus, radius/ulna, and the digits patterns. Our simulations indicate that in the proximal region (humerus, radius/ulna) the contributions of growth, reaction and diffusion are equally important to patterning, but in the distal elements (digits) the reaction and diffusion contributions are much greater than the contribution of growth to the formation of the digits. Through a single framework, we simulate the whole-limb skeletal patterns in both mice and axolotls, despite their morphological differences. These results highlight the model’s potential to explore conserved and divergent features of limb development from an evolutionary perspective through a unified mechanism across species.

## Author summary

Understanding limb skeletal patterning is a central question in developmental biology. Numerical models provide a means to explore this process. We introduce the Growth- Reaction-Diffusion (GRD) framework, which integrates tissue expansion (growth), gene regulation and cellular specification (reaction), and signaling spread (diffusion). By normalizing patterning dynamics relative to growth, the framework explores how the relative contributions of growth, reaction, and diffusion vary across the limb, influencing the timing and positioning of skeletal elements. The model successfully reproduces the whole-limb skeletal patterning in both mice and axolotls, supporting the idea of a shared underlying mechanism. This cross-species framework offers a versatile computational tool for studying fundamental processes in morphogenesis, with potential applications in developmental biology, and evolutionary studies.

## Introduction

Tetrapod limb development is an exemplary model system for exploring how developmental processes are coordinated during organogenesis. The vertebrate limb is patterned along three primary axes: the proximodistal axis (PD), which is segmented into stylopod (humerus or femur), zeugopod (radius/ulna or tibia/fibula), and autopod (hand or foot with digits); the anteroposterior axis (AP), which specifies digit number and identity [1]; and the dorsoventral axis (DV). Remarkably, despite diverging over 365 million years of evolution and exhibiting striking differences in morphology, growth strategy, and appendage function, tetrapod species share conserved skeletal patterning principles [2,3]. This conservation suggests that a common underlying developmental logic — likely involving shared molecular signaling networks and physical self-organizing mechanisms — generates the diversity of limb forms observed across vertebrates [4,5]. Numerical simulations of limb patterning have served as essential exploratory tools for understanding how this logic operates, yet most existing models address individual limb segments or species in isolation. A unified computational framework capable of simulating whole-limb skeletal patterning across species would provide a powerful means to identify what is conserved and what varies in the developmental programs underlying tetrapod limb diversity.

Two complementary theoretical frameworks have guided computational models of limb patterning. In the positional information (PI) framework, diffusible morphogens establish concentration gradients that assign spatial identity to cells, which then differentiate accordingly [6]. Two key signaling centers act as morphogen sources during limb development: the Apical Ectodermal Ridge (AER), which provides distal cues guiding PD outgrowth [7,8], and the Zone of Polarizing Activity (ZPA), which controls AP identity [1,6]. Hox gene expression provides a molecular readout of positional identity along the PD axis [9]. While positional information explains how cells acquire spatial identity, it does not readily account for how discrete, periodic skeletal elements emerge from a continuous tissue. Reaction-diffusion (Turing) systems address this gap by describing how interactions between two or more morphogens through coupled partial differential equations can spontaneously generate stable spatial patterns [10]. These two frameworks are now understood to operate together: positional cues modulate the parameters of self-organizing Turing systems, orienting and scaling the patterns they produce [11–13]. Recent work has identified candidate molecular networks — including the BMP-Sox9-Wnt loop [14] and the GDF5-NOG-pSMAD network for joint segmentation [15]— and demonstrated that these networks are highly context-dependent, with spatial location and developmental timing modulating their behavior [16].

A critical but underappreciated element of limb patterning models is tissue growth. Growth continuously changes the size and shape of the limb bud throughout development, and this affects patterning in two distinct ways: it alters the geometry on which patterns form, and it transports molecular signals through tissue expansion, introducing a convective term into the reaction-diffusion equations [17,18]. Theoretical work has established that growth can drive pattern formation in parameter regimes inaccessible to static domains [19], and that the history of growth determines which patterns emerge [20,21]. More recent work extends this analysis to non-uniform growth modes [22], and to systems where growth is coupled to morphogen concentration [23]. A key challenge in this body of work is that analyses are typically framed relative to reaction or diffusion timescales, with growth treated as slow or as a perturbation [24,25]. Yet in a developing tissue, growth is the process most directly accessible to experimental measurement (from time-lapse imaging of limb bud outlines, for example) while the timescales of molecular reaction and diffusion are far harder to quantify directly. Normalizing the reaction-diffusion system relative to the growth timescale, rather than the reverse, therefore offers a physically grounded approach that places experimentally measurable quantities at the center of the formulation. In this framework, two non-dimensional parameters naturally emerge that express the relative contributions of reaction and diffusion compared to growth, allowing direct comparison across different spatial regions and developmental stages, and enabling straightforward re-dimensionalization of model outputs into biologically interpretable units [26].

Despite these advances, existing computational models of limb skeletal patterning address individual segments or restricted subdomains rather than the limb as a whole. Models of digit patterning have provided fundamental insights into autopod self-organization but do not address the proximal skeleton [13,14,27–29]. Conversely, models of PD axis specification [30] capture the establishment of stylopod, zeugopod, and autopod identity but do not predict the spatial pattern of skeletal condensations. Models that span multiple segments typically divide the limb into discrete zones with minimal coupling between them, rather than treating the limb as a continuous and dynamically evolving field [31,32]. Cross-species comparisons have been made for specific elements, most notably the fin-to-limb transition reinterpreted as a Turing pattern reorganization [33] and digit segmentation patterns conserved between birds and mammals [15], but no unified framework has been applied to simulate whole-limb skeletal patterning simultaneously across species with substantially different growth dynamics and limb morphologies.

In this paper, we present a computational framework that integrates experimentally based geometric growth, molecular transport due to growth, and a reaction-diffusion system to simulate the formation of the entire limb. We normalize the reaction and diffusion time scales with respect to the growth characteristic time, which provides insight into the balance between growth, reaction and diffusion in the pattern formation. Within this formulation, positional information modulates the weight of reaction and diffusion relative to growth. We illustrate the influence of different weight combinations on simple growing geometries to facilitate the interpretation of the non-dimensional parameters. In realistic geometries, our model reproduces the continuous emergence of limb segments in both mice and axolotls, and also reveals how different spatio-temporal balances between growth, reaction, and diffusion influence successful whole-limb patterning.

## Materials and methods

### Growth

Biologically, growth results from a combination of cell proliferation, changes in cell size, cell migration, and production of extracellular matrix. These complex processes lead to both expansion of tissue volume and changes in tissue shape. These are approximated through an elastic deformation driven by the displacement of the limb profile. This deformation allows us to estimate a continuous growth rate from time-series images of a developing limb. As the limb outline changes shape during development, the movement of the tissue, characterized by the trajectories of material points, is calculated.

The initial position of a tissue element is defined in a two-dimensional domain using the Lagrangian coordinates $𝐗=(X_{1},X_{2})$. The position of a tissue material point is expressed by the bijective function Γ


$$\begin{array}{c} \Gamma :{ℜ}^{2}⟶{ℜ}^{2},\;𝐱=\Gamma (𝐗,t)=(\Gamma_{1}(𝐗,t),\Gamma_{2}(𝐗,t)), \end{array}$$ (1)

where **x** is the current spatial position at time *t*, with Γ(𝐗,0)=𝐗 and $\Gamma^{-1}(𝐱,t)=𝐗$. We consider the deformation of the tissue entirely due to growth, and we deduce Γ using experimental data from specific domains (see next section). The growth velocity at the current configuration is given by


$$\begin{array}{c} 𝐚(𝐱,t)=\frac{d𝐱}{dt}, \end{array}$$ (2)

where the function 𝐚(𝐱,t) is considered to be known and twice continuously differentiable. We define the scalar


$$\begin{array}{c} S(𝐱,t)=\nabla \cdot 𝐚, \end{array}$$ (3)

which measures the growth rate, i.e., the rate of area change per unit area.

#### Derivation of growth from experimental images.

**Fig 1:**
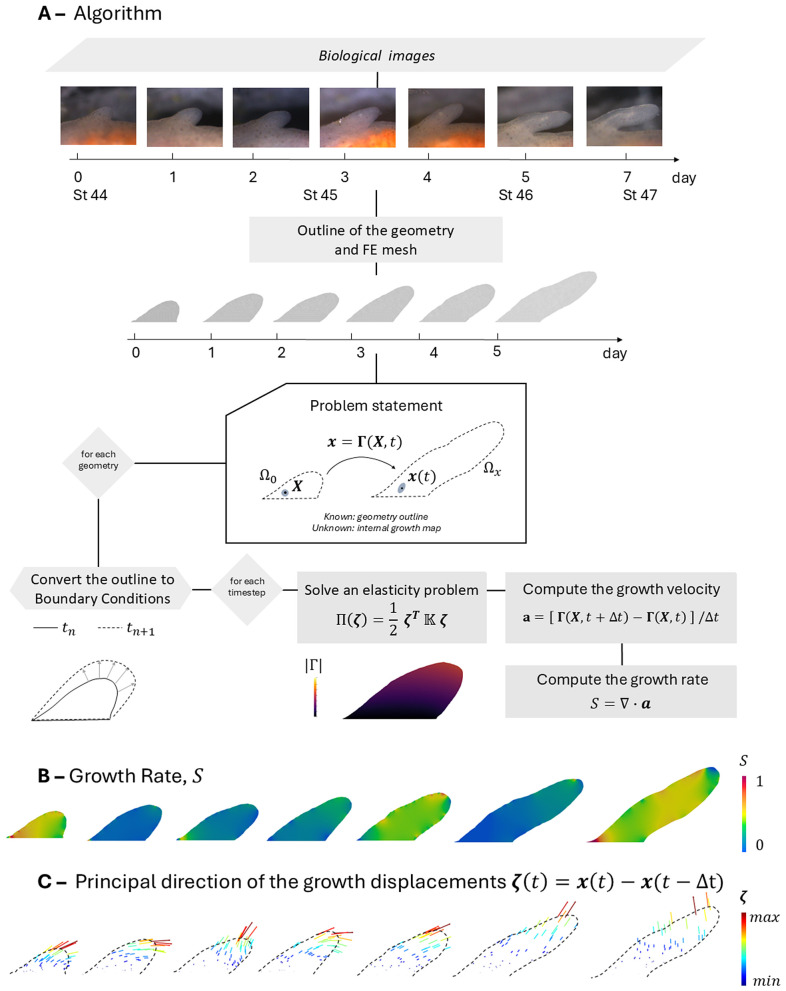
Numerical computation of growth rate from experimental images of developing axolotl limbs. **(A)** Stereo microscopy images were taken daily for a week from the same animal. An approximate correspondence between days and the axolotl limb developmental stages is provided for a practical temporal reference [34]. The limb outline was manually extracted from these images and meshed using triangular linear elements. The material points 𝐱(t) represent the current configuration $\Omega_{x}$, which is related to their initial position **X** in $\Omega_{0}$ through the mapping function Γ(𝐗,t). Each extracted outline was converted into boundary conditions, which were enforced in an elasticity problem to solve for the displacement field ζ. This displacement was then used to compute the growth velocity **a** and the growth rate *S*. **(B)** Computed growth rate *S* visualized across developmental time points, highlighting the non-uniform and dynamic nature of growth within the limb. **(C)** Principal directions of displacement vectors $\zeta_{i}$ at each node, revealing the anisotropic nature of tissue growth.

To compute the growth rate from experimental images, the growing domain of the tissue $\Omega_{x}(t)$ is treated as an elastic continuum subjected to boundary displacement conditions. By constraining the boundary $\partial \Omega_{x}(t)$ to match experimental outlines at different time points, we estimate how each point in the tissue moves over time by minimising an elastic energy function, but consider the displacements of the tissue solely due to growth. Experimental data providing the outlines of the geometry at multiple time points were used to deduce both (Fig 1A). For the mice case, the outline was provided in [35], while for the axolotl, the outline was manually extracted from images using Fiji [36].

More specifically, to compute Γ in our 2D domain discretized with finite elements [37], the quadratic elastic energy function Π(ζ) is minimized with $\zeta =[\zeta_{1},\ldots ,\zeta_{N}]$ being the displacement in all the *N* nodes of the mesh, with $\zeta_{i}(t)={𝐱}_{i}(t)-{𝐱}_{i}(t-\Delta t)$,


$$\begin{array}{c} \Pi (\zeta )=\frac{1}{2}\;\zeta^{T}\;𝕂\;\zeta \;, \end{array}$$ (4)

where 𝕂 is the growth rigidity matrix, which characterizes how tissue responds to boundary expansion. In the finite element implementation, it corresponds to the standard stiffness matrix. We assume a constant, homogeneous, and isotropic growth rigidity, i.e., a constant and homogenous Young modulus *E* and Poisson's ratio ν. This means we treat all regions of the tissue as equally responsive to growth. We recognize that this a simplification, as developing limbs may exhibit spatial variations in proliferation rates and growth directions [38]. The minimization process determines how tissue expansion distributes most evenly throughout the growing domain while maintaining tissue continuity. Despite assuming homogeneous growth rigidity, this approach generates growth rate patterns that are non-homogeneous, anisotropic, and dynamic over time for both the axolotl and mouse (Figs 1B, 1C and S1A).

We use as many geometries as the number of time steps. To obtain the outline for each time step, the geometry is linearly interpolated between experimental data points (Fig 1A). Then, Π(ζ) is minimized for each time step to deduce growth displacement, ζ(t), which can be related to the growth velocity as


$$\begin{array}{c} 𝐚(𝐱,t)=\frac{\zeta (t)-\zeta (t-\Delta t)}{\Delta t}, \end{array}$$ (5)

where ζ(t−Δt) is the displacement at the previous time step, and Δt the time step size.

### Reaction-diffusion equation on a growing domain

A dimensional version of the reaction-diffusion (RD) equation in a growing domain [17] is given by


$$\begin{array}{c} \frac{d𝐜}{dt}+S(𝐱,t)\;𝐜=𝔻\;\nabla_{x}^{2}𝐜+𝐑(𝐜), \end{array}$$ (6)

where $\nabla_{x}^{2}$ denotes the Laplacian operator applied to each component of concentrations in 𝐜=[u(𝐱,t),v(𝐱,t)]. The total time derivative d𝐜/dt can be decomposed as d𝐜/dt=∂𝐜/∂t+𝐚·∇𝐜, with the first term representing changes for fixed spatial points **x**, and the second term accounts for advective transport of *u* and *v* due to tissue motion, represented by the velocity field 𝐚(𝐱,t). The convective term S(𝐱,t)𝐜, with S=∇·𝐚 (see equation 3), accounts for local expansion or compression of the tissue, which effectively dilutes or concentrates *u* and *v*. The term $𝔻\;\nabla_{x}^{2}𝐜$ represents the diffusion process, i.e., the transmission of specification information across the tissue. This can occur through the diffusion of signaling molecules or through direct cell-cell communication mechanisms, such as gap junctions or mechanical coupling. The diffusion tensor 𝔻 is assumed diagonal, with entries $D_{u}$ and $D_{v}$ corresponding to the diffusion coefficient of *u* and *v*, respectively, implying that no cross-diffusion is considered. Vector **R**(**c**) corresponds to the reaction term with an activator-substrate kinetics, specifically Schnakenberg kinetics [39],


$$\begin{array}{c} 𝐑(u,v)=[\begin{array}{c} k_{1}-u+u^{2}v\\ k_{2}-u^{2}v \end{array}]. \end{array}$$ (7)

**Fig 2:**
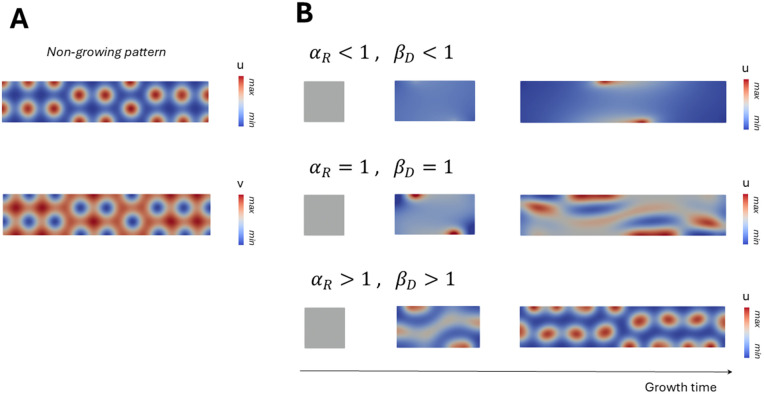
Growth-reaction-diffusion system. **(A)** Non-growing rectangle ($\alpha_{R}>1$, $\beta_{D}>1$) visualized for both *u* and *v*, demonstrating the out of phase pattern. **(B)** Simulated patterns on a growing rectangle for varying $\alpha_{R}$ and $\beta_{D}$. Results for an initial, intermediate and final domain size are shown for the variable *u*. Comparison with non-growing domain can be done for ($\alpha_{R}>1$, $\beta_{D}>1$).

The reaction term represents the cellular mechanisms by which specification information is interpreted and acted upon. This may include transcriptional activation of genes, signal transduction cascades, or other intracellular processes that regulate cell fate decisions. The variable v(𝐱,t) acts as a substrate that is spatially out of phase with the activator u(𝐱,t) (Fig 2A), such that regions of high *u* coincide with regions of low *v*, and vice versa. Schnakenberg kinetics were chosen as a well-established and widely used non-linear instance of activator-substrate dynamics, though other out-of-phase reaction terms could serve as suitable alternatives. Within the context of our work, the variable *u* represents the specification of the skeletal elements, i.e., differentiation to cartilage. High values of *u* simulate *Sox9*-positive cells, whereas high values of *v* correspond to *Sox9*-negative cells. Consequently, the patterns of *u* generated in a forelimb model correspond to the skeletal elements: humerus, radius, ulna, and digits, which will be the focus of visualization for the remainder of this paper.

#### Definition of the timescales.

Each process governing pattern formation, growth, diffusion, and reaction, operates on a characteristic timescale, and the relative magnitudes of these timescales determine their contributions to the resulting pattern. For clarity, we denote in the sequel by $\left(c_{1},c_{2}\right)$ variables (*u*, *v*), and $R_{1},R_{2}$ the corresponding reaction terms of vector **R** in (7). We define *L* as the characteristic length, which we choose to be the initial length of the domain, *L*_0_. The reaction rate ω, characteristic of the kinetic scheme, is used to non-dimensionalize the reaction term **R**. The diffusivity of each chemical component is denoted as $D_{i}$, and we define $D_{max}=\max\{D_{i}\}$. The non-dimensionalized diffusivity is introduced as ${\bar{D}}_{i}=D_{i}/D_{max}$.

Two timescales corresponding to the two elements on the right of (6), $T_{D}$ and $T_{R}$ for diffusivity and reaction kinetics, respectively, are introduced:


$$\begin{array}{c} T_{D}=\frac{L_{0}^{2}}{D_{1}}\;\text{,}\;T_{R}=\frac{1}{\omega }. \end{array}$$ (8)

Here, the characteristic timescale $T_{D}$ represents the time it takes for diffusion to spread across a distance equal to the characteristic length scale *L*_0_. The timescale $T_{R}$ refers to the reference duration of a chemical reaction.

The characteristic timescale $T_{G}$, which is associated with domain expansion due to growth, is defined as


$$\begin{array}{c} T_{G}=\frac{L_{0}}{a_{c}}, \end{array}$$ (9)

where the characteristic growth velocity


$$\begin{array}{c} a_{c}=\frac{L_{f}-L_{0}}{t_{f}} \end{array}$$ (10)

has been defined. Here, $t_{f}$ corresponds to the final time, at which the domain reaches the final length $L_{f}$, and $a_{c}$ represents the characteristic velocity at which the domain grows from its initial to its final geometry. Rewriting the growth timescale in terms of the final apparent strain $\varepsilon_{f}=(L_{f}-L_{0})/L_{0}$ and using the relation $a_{c}=\varepsilon_{f}L_{0}/t_{f}$ in (9) results in $T_{G}=t_{f}/\varepsilon_{f}$. This expression provides an alternative interpretation for $T_{G}$: the time required to achieve a net 100% strain in the domain. As described above, domain growth is modeled through an imposed displacement at the boundaries, computing the interior deformation that is compatible with the displacements by minimizing the energy Π defined in (4). The resulting deformation is assigned to a growth strain, i.e., neglecting any elastic deformation.

#### Non-dimensionalization.

The process of non-dimensionalization of the RD equations captures the interactions and interdependencies within the system by identifying the key dimensionless groups that govern the system. Any of the three timescales, $T_{G}$, $T_{R}$ or $T_{D}$, could be used to non-dimensionalize the time variable. Because the characteristic time of growth is the only experimentally measurable quantity in our problem, we use it to non-dimensionalize the time variable. Introducing $\tau =t/T_{G}$ into (6), and writing the equation in component form for clarity, we obtain


$$\begin{array}{c} \frac{\partial {\bar{c}}_{i}}{\partial \tau }+\bar{𝐚}\cdot \nabla_{\bar{x}}{\bar{c}}_{i}+{\bar{c}}_{i}\;\bar{S}=\frac{T_{G}}{T_{D}}{\bar{D}}_{i}\nabla_{\bar{x}}^{2}{\bar{c}}_{i}+\frac{T_{G}}{T_{R}}{\bar{R}}_{i}(\bar{c}),\;i=1,2 \end{array}$$ (11)

where $\bar{c_{i}}=c_{i}/C_{i}$ and $\bar{𝐱}=𝐱/L_{0}$, with $C_{i}$ the reference concentration for the *i*^th^ chemical component. The non-dimensional growth rate is $\bar{S}=\nabla_{\bar{x}}\cdot \bar{a}$ with $\bar{a}=a/a_{c}$ and $a_{c}=L_{0}/T_{G}$. The non-dimensional time τ can be related to the apparent strain as


$$\begin{array}{c} \tau =\frac{t}{T_{G}}=\varepsilon_{f}\frac{t}{t_{f}}. \end{array}$$ (12)

Dropping the bars and reverting τ back to a non-dimensional *t* to simplify notation, results in


$$\begin{array}{c} \frac{\partial c_{i}}{\partial t}+𝐚\cdot \nabla_{x}c_{i}+c_{i}\;S=\beta_{D}D_{i}\nabla_{x}^{2}c_{i}+\alpha_{R}R_{i}(𝐜),\;i=1,2 \end{array}$$ (13)

where we have been introduced the parameters $\beta_{D}$ and $\alpha_{R}$, defined by


$$\begin{array}{c} \beta_{D}=\frac{T_{G}}{T_{D}}=\frac{D_{\max}T_{G}}{L_{0}^{2}}\;\text{and}\;\alpha_{R}=\frac{T_{G}}{T_{R}}=T_{G}\;\omega . \end{array}$$ (14)

One advantage of this approach is that the explicit knowledge of $T_{G}$ enables straightforward conversion of nondimensional results back into dimensional units, allowing direct comparison with experimental data. The system in (13) can be expressed in compact form for the two concentrations as


$$\begin{array}{c} \frac{d𝐜}{dt}+S(𝐱,t)\;𝐜=\beta_{D}𝔻\;\nabla_{x}^{2}𝐜+\alpha_{R}𝐑(𝐜). \end{array}$$ (15)

#### Interpretation of the parameters $\alpha_{R}$ and$\beta_{D}$.

The parameters $\alpha_{R}$ and $\beta_{D}$ serve as indicators of the relative importance of reaction and diffusion compared to growth.

When $\alpha_{R}$ and $\beta_{D}$ are less than one, growth dominates. In this regime, the pattern is strongly shaped by the growth, through the convective term S𝐜, which represents the transport of *u* and *v* caused by tissue expansion due to growth as shown in the supplementary information (S1B, S2A and S3A Figs). When both parameters are close to one, all processes, reaction, diffusion, and growth, proceed on comparable timescales, yielding patterns shaped by the interplay of all mechanisms. When $\alpha_{R}$ and $\beta_{D}$ exceed one, reaction and diffusion dominate over growth. The spatial pattern is governed primarily by the intrinsic properties of the reaction–diffusion system, and growth has negligible influence, producing outcomes similar to those observed in static (non-growing) domains (Fig 2B). Note that the inclusion of the convective term S𝐜 is essential when growth dominates reaction and diffusion effects (see S1C, S2B and S3B Figs).

To illustrate these effects, we have simulated numerically an initially squared domain that is stretched along the horizontal direction, as shown in Fig 2. When $\alpha_{R},\beta_{D}>1$, the patterns obtained are similar to those when a static domain with different increasing sizes are modelled, resulting in a sequence of pattern bifurcations, with a negligible contribution of the convective terms. Instead, when $\alpha_{R},\beta_{D}<1$, growth effects dominate and produce an apparent stretching of the patterns. For intermediate values, stretching and pattern bifurcations are combined as the domain grows.

In practical terms, these parameters provide a framework for interpreting and estimating dimensional timescales. For example, in axolotl limb development, experimental data indicate a final apparent strain $\varepsilon_{f}=3.95$ over a period of 12 days. This implies a characteristic growth timescale $T_{G}=t_{f}/\varepsilon_{f}\approx 3.03$ days. Based on the range of $\alpha_{R}$ values used in our simulations (from 1 to 30), the corresponding reaction timescales $T_{R}=T_{G}/\alpha_{R}$ span from 3.03 days down to 0.101 days. Similarly, the diffusion timescales $T_{D}=T_{G}/\beta_{D}$, with $\beta_{D}$ up to 15, span from 3.03 days down to 0.202 days. These estimates demonstrate how the non-dimensional framework allows re-dimensionalization of the system and facilitates interpretation of quantities of interest across biological contexts.

### Numerical simulations

The governing equation (15) is discretized in space using the finite element method and in time with the implicit midpoint rule. The numerical implementation is performed in MATLAB [40] in an in-house code. Linear triangular elements with three-point Gauss quadrature are used to mesh the domain. According to our non-dimensionalization, relative time is given by $\tau =\varepsilon_{f}t/t_{f}$, and therefore at final time $\tau_{f}=\varepsilon_{f}$. In our simulations, the number of time increments is given by $\varepsilon_{f}/\Delta \tau$, with Δτ the non-dimensional time step. In the mice case, we used Δτ=0.0017 and a total number of increments of 3804 to achieve the corresponding $\varepsilon_{f}\sim 6.5$ fold lengthening. Homogeneous Neumann boundary conditions are applied. The function **R** follows Schnakenberg kinetics [39] with model parameters *k*_1_, *k*_2_, and *d* that ensure pattern formation (refer to S1 Table for the parameters used and section S1 Text for the parameter space).

**Fig 3:**
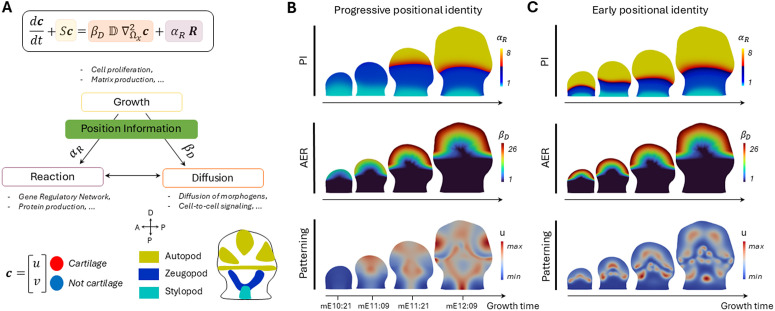
Skeletal patterning of a representative growing mouse limb bud simulated withe the GRD computational framework. (**A**) Vector **c** corresponds to the two out-of-phase concentrations, *u* and *v*, where high values of *u* indicate differentiation of skeletal elements through cartilage condensation. The convective term S𝐜 captures the effect of growth, with *S* representing the growth rate. The diffusion term is $\beta_{D}𝔻\nabla_{x}^{2}𝐜$, and the reaction term is $\alpha_{R}𝐑(𝐜)$. $\alpha_{R}$ and $\beta_{D}$ indicate the contribution of reaction and diffusion relative to growth in the system. Growth, reaction and diffusion refer to tissue expansion, information regulation and signaling communication. The mice limb is oriented along the PD/AP axis. (**B**) The first row shows the distribution of $\alpha_{R}$, informed by PD Positional Information (PI) patterns. The second row displays the input map distribution of $\beta_{D}$, informed by signaling from the Apical Ectodermal Ridge (AER). The final row shows the simulated skeletal patterning, with regions of high *u* values shown in red indicating locations of cartilage condensation. The humerus, followed by the radius and ulna, and, finally, five digits can be identified. (**C**) The distribution of $\alpha_{R}$ and $\beta_{D}$ are changed to represent early positional identity. This results in incomplete patterning. Our model suggests that the identity of the three limb segments (stylopod, zeugopod, and autopod) occurs sequentially (Fig 3B) and is not predetermined at the limb bud stage.

**Fig 4:**
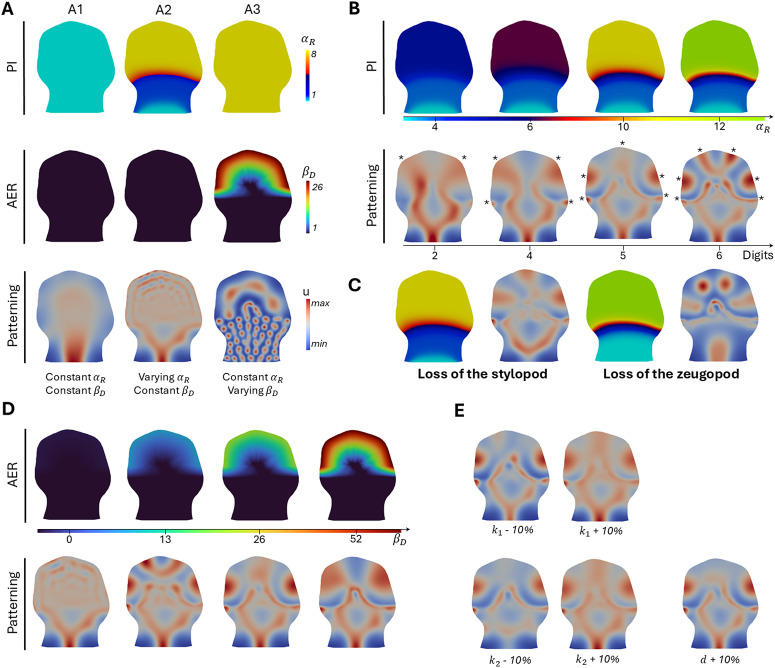
Modifying positional cues and simulating limb segment outcomes with the GRD framework. The final PI, AER and skeletal pattern distribution is represented. (**A**) A1 represents the framework without incorporating PI or AER, implemented as constant and homogeneous $\alpha_{R}$ and $\beta_{D}$, resulting in a simple stripe pattern. A2 includes PI by allowing spatial variation in $\alpha_{R}$ but excludes AER by keeping $\beta_{D}$ constant and homogeneous. This preserves the proximal pattern seen in Fig 3B but disrupts stripe orientation. A3 incorporates AER by using a spatially varying $\beta_{D}$ but excludes PI with constant $\alpha_{R}$, leading to the loss of more proximal skeletal elements. (**B**) Increasing the strength of the reaction relative to growth (controlled by the parameter $\alpha_{R}$) in the autopod region leads to a greater number of digits. The stars indicate the digits. (**C**) Modifying the spatial boundaries of the stylopod or zeugopod regions using the values of $\alpha_{R}$ can result in the loss of proximal skeletal limb segments. (**D**) Decreasing the strength of the diffusion relative to growth (controlled by the parameter $\beta_{D}$) leads to a loss of the digits orientation. (**E**) Sensitivity analysis on the kinetic parameters *k*_1_ and *k*_2_ of the Schnakenberg reaction term, and on the diffusivity ratio *d*. The stylopod and zeugopod patterns remain relatively unaffected compared to the autopod, which is expected given the stronger influence of reaction-diffusion dynamics on autopod patterning. Overall, the patterns show robustness to these parameter variations.

**Fig 5:**
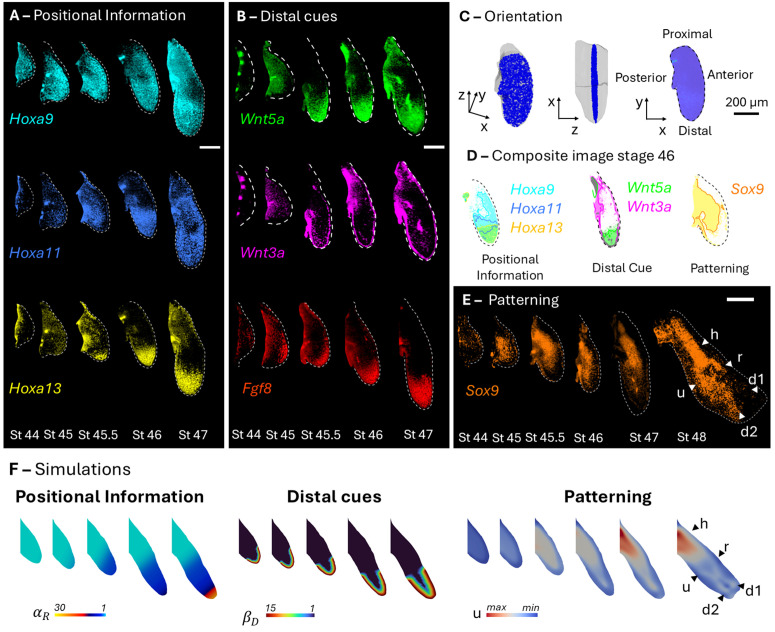
Experimental imaging and computational simulation with the GRD model of axolotl limb patterning. (**A**) Fluorescence imaging of *Hoxa9* (top row), *Hoxa11* (middle row), and *Hoxa13* (bottom row) at successive stages of axolotl limb development. These genes serve as markers of proximal-to-distal PI, and inform the spatiotemporal map of $\alpha_{R}$ in our simulations (**F**, left, “Positional Information”). (**B**) Fluorescence imaging of *Wnt5a* (top row), *Wnt3a* (middle row), and *Fgf8* (bottom row) key markers of the distal cue, used to inform the spatiotemporal map of $\beta_{D}$ in our simulations (**F**, center, “Distal cues”). (**C**) Orientation reference for the 2D plane (in blue) from the 3D limb imaging (in gray) shown in panels A, B, and E. The 2D domain is oriented along the proximal-distal (PD) and antero–posterior (AP) axes. (**D**) Composite representation of positional information (from *Hoxa* genes), distal signaling cues (from Wnt genes), and skeletal patterning at developmental stage 46. Note: *Hoxa9*, *Hoxa11*, and *Hoxa13* are shown in cyan, blue, and yellow respectively; their co-expression results in mixed colours, such that regions where *Hoxa13* overlaps with *Hoxa9* and *Hoxa11* appear green. (**E**) Fluorescence imaging of *Sox9*, a marker of chondrogenic progenitors, across developmental stages. At stage 48, skeletal elements are labeled: h (humerus), r (radius), u (ulna), d1 (first digit), d2 (second digit). (**F**) The GRD model simulation results on the experimental limb geometry. The inputs $\alpha_{R}$ and $\beta_{D}$ are informed by gene expression data (**A** and **B**); the output variable *u* simulates the spatial pattern of skeletal condensations (**E**).

Simulations were initialized with random perturbations (10% variation) around a steady state defined by **R**(**c**) = **0**. For mouse and axolotl limb models, an epithelial layer was included at *t* = 0 with **c**(0) = **0** (Figs 3–5). The effect of this initial condition is further explored in S4 Fig.

### HCR-FISH and whole-mount hybridization imaging

Hybridization Chain Reaction Fluorescence In Situ Hybridization (HCR-FISH) was performed in whole-mount developing limb following the protocol provided by Molecular Instruments and as described by [41], without modification. Tissues were fixed in 4% paraformaldehyde, dehydrated in a methanol series, and stored at -20°C until use. Rehydrated tissues were treated with proteinase K, post-fixed, and hybridized with probes overnight at 37°C. After probe washes, HCR amplification was carried out using snap-cooled hairpins, followed by mounting in 1.5% low-melting agarose in glass capillaries and refractive index matched in *EasyIndex* (*LifeCanvas Technologies*).

Light-sheet imaging was performed on a Zeiss Z.1 microscope at 20X with dual side illumination. Refractive index matched, agarose-embedded samples were imaged in *EasyIndex*. Image stacks were denoised in Zen Blue, and post-processing (rotation, cropping, and brightness/contrast adjustment) was conducted in Fiji [36] and MATLAB [40].

## Results

### Simulation of the whole limb mouse patterning with the GRD framework and sensitivity analysis

To explore how the GRD framework captures skeletal patterning during limb morphogenesis, a classical model organism in developmental biology is used: the mouse (Fig 3). The shape of the mouse limb bud during growth was extracted from outlines of the limb bud from stage mE10:21 to mE12:09 [35]. The GRD framework is able to replicate the sequential appearance of the skeletal pattern by increasing $\alpha_{R}$ along the PD axis, reflecting the PI conveyed by *Hoxa* gene expression [42]. Higher values of $\beta_{D}$ in distal regions compared to the proximal region capture aspects of signaling associated with the AER [43]. Though not direct representations of gene expression or tissue structures, these parameters act as spatial modulations within the model that align with known biological patterning influences (Fig 3B). As the limb bud grows and changes shape over time, these spatial variations in $\alpha_{R}$ and $\beta_{D}$ become more pronounced. An increase in $\alpha_{R}$ from proximal to distal suggests that the reaction contributes more to patterning, relative to growth, in distal segments (Fig 3B). Similarly, an increase in $\beta_{D}$ toward the distal tip of the limb bud, indicates that diffusion likewise gains relative weight over growth in that region (Fig 3B).

The GRD framework provides a tool to investigate how the temporal evolution of positional identity affects patterning outcomes, allowing us to compare two classic theoretical models for the establishment of positional identity: progressive positional identity (Fig 3B) and early positional identity (Fig 3C) [44]. The progressive model suggests that positional identity is acquired sequentially, resulting in the stylopod determined first, followed by the zeugopod, and finally the autopod [45]. To simulate this scenario, $\alpha_{R}$ evolves from a single uniform value into two and then three distinct regions using a sigmoid function normalized to the final domain length. The different regions correspond to the limb segments and reflect the gradual establishment of positional identity. Similarly, $\beta_{D}$ is modulated by linearly increasing its distribution over time at the distal tip. In contrast, in the early positional identity model, both $\alpha_{R}$ and $\beta_{D}$ distributions and values are fixed from the limb bud stage and are stretched with the domain growth (Fig 3C). The progressive identity scenario leads to appropriate skeletal patterning, with sequential emergence of the humerus, radius/ulna, and digits. These results support a model in which positional identity unfolds progressively during development, rather than being fully established at early stages.

To explore the sensitivity of the model to $\alpha_{R}$ and $\beta_{D}$, we systematically modified these parameters (Fig 4A-4D). In the absence of changes to $\alpha_{R}$ and $\beta_{D}$, simulating lack of positional information and lack of an AER, the model simulates simple stripe patterns, potentially corresponding to a rudimentary stylopod element (Fig 4A1). Including PI (changes in $\alpha_{R}$) but not the proximal-distal cues of the AER ($\beta_{D}$) resulted in circumferential, rather than radial, elements in the digits (Fig 4A2). Including AER ($\beta_{D}$) but excluding PI (changes in $\alpha_{R}$), produces spots, rather than stripes, in the proximal skeletal elements (Fig 4A3).

These sensitivity analyses suggest that $\alpha_{R}$ influences the wavelength of the pattern, which determines the number of stripes (skeletal elements) formed. In the typical case, this allows a transition from one stripe (humerus) to two stripes (radius and ulna) and eventually to multiple stripes (digits). As the value of $\alpha_{R}$ increases at the autopod, more digits are formed (Fig 4B). Increasing $\alpha_{R}$ from 4 to 12 increases the number of digits from 2 to 6. Even though PI is defined along the proximal-distal axis through the spatial modulation of $\alpha_{R}$, the resulting pattern also reflects anterior-posterior organization of the number of skeletal elements. This highlights how PD inputs can influence AP patterning outcomes and reinforces the known interdependence between the two axes [46,47].

To further explore $\alpha_{R}$ (the relative weight of reaction to growth) in segment identity, we tested whether individual limb segments could be selectively removed or shortened by shifting the transition points between the different $\alpha_{R}$ values (cyan, blue and yellow regions in Fig 4C). We applied this to both the stylopod and the zeugopod by shifting the transition point from the cyan region to the blue one. In both cases, shortening a segment is achievable, consistent with biological experiments [48], but it simultaneously alters autopod patterning. The framework therefore cannot completely remove or shorten the stylopod or zeugopod without affecting the autopod, reflecting the interconnected nature of limb segments where changes to one region impact the development of others.

Parameter $\beta_{D}$ (the relative weight of diffusion to growth) affects the orientation of the radial stripe-like pattern of the digits. With a progressive increase in $\beta_{D}$, the pattern gradually orients radially (Fig 4D), with higher values producing a more ordered digit-like arrangement. This highlights the critical role of $\beta_{D}$ in influencing digit patterning, similar to the AER [49,50].

A sensitivity analysis on the kinetic parameters *k*_1_ and *k*_2_ of the Schnakenberg reaction term and on the diffusivity ratio *d* is provided in Fig 4E. The stylopod and zeugopod patterns remain relatively unaffected compared to the autopod, which as expected, is more strongly influenced by reaction-diffusion dynamics. The patterns overall show robustness to these parameter variations.

### Integrating experimental data to simulate axolotl limb patterning

To test the robustness of the GRD framework across divergent tetrapod species, we applied it to the axolotl salamander (Ambystoma mexicanum), an anamniote with well-documented differences in limb development compared to amniotes such as mice and chickens [51]. While several features of their development are unique, key principles are conserved [52–55].

One of the most striking differences is that salamanders do not form a morphologically distinct AER. Despite this, the region retains its function to induce distal outgrowth [54]. Molecular expression also diverges in some aspects. For instance, while *Ffg8* is restricted to the AER in mice and chickens, in axolotls it is only expressed in the mesenchyme [56–58]. Another key difference lies in digit formation. In amniotes, digits form from postaxial to preaxial; through a paddle stage that is later sculpted by apoptosis in the interdigital regions [59]; while in axolotl they emerge in the opposite order through localized outgrowth [51,59]. Despite these differences the fully patterned limb is remarkably similar. This raises the question whether the same underlying principles drive patterning in both systems, even if the specific outcomes may vary.

To inform the computational model, we imaged key genes involved in axolotl limb patterning at different developmental stages (stages 44–47 [34]) using light-sheet fluorescence microscopy (Fig 5A, 5B, 5E). Whole-mount samples were imaged in 3D, reoriented, and a representative 2D section capturing spatial gene expression patterns along the PD and AP axes was extracted for analysis (Fig 5C). Fig 5A shows the expression of the *Hox* genes *Hoxa9*, *Hoxa11*, and *Hoxa13*, which encode PI along the PD axis of the developing limb [52]. These markers broadly correspond to future stylopod (*Hoxa9*), zeugopod (*Hoxa11* and *Hoxa9*), and autopod (*Hoxa13*, *Hoxa11* and *Hoxa9*) regions. We observed that *Hoxa9* was expressed broadly throughout the mesenchyme from stage 44 onward. *Hoxa11* was expressed from the middle portion of the limb bud to the distal tip from stage 45 to stage 47. *Hoxa13* first appeared at the distal tip around stage 45.5 and remained restricted to the autopod region through subsequent stages. These results are consistent with previously reported spatiotemporal expression patterns of *Hoxa* genes during axolotl limb development [60]. Additionally, we imaged mRNA expression associated with distal cues including *Wnt3a*, *Wnt5a*, and *Fgf8* (Fig 5B) [56–58]. *Wnt3a* expression is localized to the limb epithelium. *Wnt5a* is expressed in both the epithelium and the mesenchyme at the distal tip. *Fgf8* is first detected at stage 45 in the distal mesenchyme and overlaps spatially with *Wnt5a*, though it emerges slightly earlier. All channels are shown together in composite images in Fig 5D. The presence of similar expression domains in axolotls supports the idea that key signaling pathways are conserved, even in the absence of a morphologically distinct AER [61].

In Fig 5E, we imaged the expression of *Sox9*, a transcription factor marking chondrogenic progenitors. By stage 48, distinct skeletal elements become visible, including the humerus (h), radius (r), ulna (u), and digits (d1, d2). Earlier stages (44–47) show the gradual emergence of these elements, with the humerus visible by stage 45-45.5 and the radius and ulna beginning to appear by stages 46–47. We focused our imaging and numerical analysis up to stage 48, when two of the four digits are present, as this stage is sufficient to provide a spatial reference to validate the GRD model simulations. Later stages were not included, as they follow the same principles through sequential digit addition.

Using the experimentally informed geometries and spatial inputs, we ran numerical simulations of the GRD framework to predict sites of skeletal patterning across developmental stages (Fig 5F). In these simulations, $\alpha_{R}$ and $\beta_{D}$ were assigned to reflect the distributions of *Hox* gene expression and distal signaling factors, respectively. Similar to the images in Fig 5A, the segments’ identities are acquired progressively: the stylopod region (cyan) is established from the start, followed by the emergence of the zeugopod domain (blue) around stage 45.5, and finally the autopod (orange) around the end of stage 46 to stage 47. Distal cues are applied at the distal tip from as early as stage 44–45 and persist throughout the limb development. The conversion from experimental data (Fig 5A, 5B) to computational inputs (Fig 5F) therefore relies on qualitatively matching the spatiotemporal profiles of $\alpha_{R}$ and $\beta_{D}$ (the relative weight of reaction and diffusion to growth) to the observed gene expression domains. The resulting output variable, *u*, represents the simulated skeletal elements. The model recapitulates the progressive emergence of distinct limb segments, beginning with a central condensation corresponding to the stylopod around stage 45.5, which later bifurcates into two elements by stage 46–47, followed by a second bifurcation that gives rise to additional elements aligned with digit positions observed in *Sox9* expression at stage 48. The spatial correspondence between the simulation output (Fig 5F, right) and experimental marker expression (Fig 5E) demonstrates that the underlying developmental logic captured by the GRD model is conserved and extensible beyond mouse limb development. The role of AER signaling and PI, which in our framework reflect the relative contribution of diffusion and reaction with respect to growth, is further explored in supplementary simulations (S5 Fig).

## Discussion

### Growth, Turing patterns and positional information in whole limb patterning

In this work, we introduce the Growth-Reaction-Diffusion (GRD) framework to provide a numerical tool for simulating patterning during limb formation. It is structured around three fundamental factors: 1) growth, which captures changes in size and shape; 2) the reaction, which includes gene regulatory interactions and their effects; and 3) the diffusion, encompassing morphogen signaling, cell-cell signaling, morphogen diffusion, etc. (Fig 3A).

In vivo, these mechanisms operate through gene regulatory networks which represent the network of genes that determine cell fate [62]. For digit patterning specifically, researchers have identified several promising molecular candidates that can be modeled through RD systems, including the Sox9-Bmp-Wnt feedback loop [14,29] and the TGF-β-Bmp loop [63]. Similar to previous models that assume that positional identity directly modifies molecular kinetics, our approach alters the weight of reaction and diffusion relative to growth using positional identity [13,14]. The spatial and temporal continuity allows a systematic investigation of how different segments of the limb interact, respond to perturbations, and influence one another over time (Fig 4B, 4C). We assess the removal and modification of the AER and PD cues and demonstrate altered patterning (Fig 4A). This perspective also allows to explore the timing of positional identity emergence (Fig 3B, 3C).

In the GRD framework, we use growth as the reference timescale, rather than reaction or diffusion, as in previous models [18,64]. We chose this approach because growth is an observable process that can be quantified, whereas the timescales of reaction and diffusion are more difficult to directly measure. These timescales can be understood as indicating which aspect of the system —growth, reaction, or diffusion —plays the dominant role at a given point in space and time. In our simulations the relative timescales, represented by $\alpha_{R}$ and $\beta_{D}$, vary spatially over time.

Proximally, $\alpha_{R}=1$ and $\beta_{D}=1$, indicating that reaction, diffusion, and growth all contribute to the emerging pattern. Distally, however, higher values of $\alpha_{R}$ and $\beta_{D}$ (8 and 26) indicate that reaction and diffusion, respectively, dominate over growth. This supports the assumption that, during autopod patterning, growth occurs on a slower timescale than the reaction and diffusion, and can therefore be neglected in the reaction-diffusion equations [24]. In contrast, the lower values observed in the zeugopod and stylopod suggest that growth is more tightly integrated into the patterning process in these regions. This spatial variation emphasizes the need to explicitly model growth through the convective term when addressing the entire limb patterning. As illustrated in an initially hypothetical squared domain (Figs 2A, 2B and S2A), the growth rate distribution can significantly influence the pattern when $\alpha_{R}$ and $\beta_{D}$ are low, while higher values yield outcomes similar to those on static domains or on growing domains without the convective term.

While this framework highlights the importance of growth, its current formulation has limitations. First, the use of a quadratic energy function for retrieving the bulk growth does not fully replicate the nonlinearities of this process [65]. Moreover, the model assumes no intrinsic directionality in growth and no built-in regional biases, meaning all parts of the domain are treated as equally permissive to growth. Despite this simplified starting assumption, the GRD model generates anisotropic and inhomogeneous growth rate patterns as a result of the boundary conditions (Fig 1B, 1C). However, studies have shown that growth in the developing limb has inherent directional biases and regional variations [38,66–68]. Our preliminary tests with different growth rate distributions show that the specification pattern is significantly impacted by these, both on the rectangular domain (S2 and S3 Figs) and on the developing mouse limb (S1 Fig). These results further emphasize the necessity of including the convective term in the equations, particularly in early stages where the growth dynamics are equally as important as the RD system, and the importance of incorporating biologically realistic growth dynamics in future work.

Moreover, since our model is two-dimensional, it does not account for patterning along the dorsal-ventral axis, meaning that spatial interactions in the third dimension are entirely missing from the model. In addition, future work could integrate a more detailed understanding of growth to better align with biological reality. Growth, patterning, and cellular differentiation are inextricably linked during development [69], yet the current GRD framework does not fully capture the complexity of their interactions. In particular, growth is computed independently of the R-D system, though a coupling between them should emerge, especially when the parameters $\alpha_{R}$ and $\beta_{D}$ are close to one. A particularly promising avenue would be to couple the model self-consistently. One possible approach would be to use the resulting patterns to inform local tissue rigidity, which in turn feeds back into the growth and patterning computations. Exploring this feedback loop between pattern formation and tissue growth could provide interesting insights into the mechanisms underlying limb morphogenesis.

### The GRD framework from an evolutionary perspective

Limb development follows conserved patterning principles across tetrapods, yet morphological outcomes vary significantly between species. These shared developmental mechanisms, including conserved self-organizing principles, suggest that a common morphogenetic logic underlies limb formation across tetrapods [2]. The GRD approach provides a framework to model these core processes by focusing on how spatial and temporal dynamics generate variability within a unified developmental model.

The GRD framework enables us to investigate homologous patterning mechanisms across species with different limb morphologies, such as amniotes and anamniotes (Figs 3B, 5F). Previous studies have explored developmental variations by modifying spatial and temporal cues, focusing on a specific skeletal elements (e.g., digits) or a collection of discrete zones, such as active and frozen regions, with minimal coupling between them [31]. In contrast, the GRD model treats the whole limb as a continuous field. We can reproduce known changes in digit number (Fig 4B) and, like existing studies, potentially link them to molecular regulators such as *Hox* genes [13]. However, the sensitivity of patterning to positional cues highlights the need for a more accurate spatial representation of these variations in the model (Figs 4C and S6). Specifically, this would involve improving how imaging data is translated into model inputs to better capture the spatial variations in $\alpha_{R}$ and $\beta_{D}$ distributions, allowing for more precise simulations of patterning outcomes [70,71].

Beyond capturing developmental variability, the GRD framework provides a tool for exploring evolutionary shifts in limb morphology. The evolutionary trajectories of mice and axolotls diverged over 365 million years, leading to distinct growth and patterning strategies. Yet, despite these differences, they can be studied within the same framework, enabling us to identify shared principles across tetrapods and explore a range of evolutionary changes. For example, features such as autopod elongation in bats [72], digit loss in tetrapods [4] or even limb regeneration properties [73] could be reexamined as a modulation of growth rate relative to reaction and diffusion. One of the most striking evolutionary transitions, the fin-to-limb shift, offers a compelling test case for this model [33]. In addition to studying changes like those in the AER, the GRD framework provides a complementary perspective: could modifications in growth rate have also contributed to key structural changes? The GRD model serves not only as a simulation tool but also as a means to guide future experimental work, potentially revealing the molecular pathways underlying these transitions.

In summary, this study applies the Growth-Reaction-Diffusion (GRD) framework to investigate how growth dynamics influence patterning in limb development. This perspective allows reinterpreting the interplay between RD systems and positional identity, offering a shared self-organizing mechanism for all limb segments. Beyond its developmental applications, the framework opens new avenues for exploring evolutionary transitions. Future work will extend the model to include realistic anisotropic inhomogeneous growth to further bridge the gap between theoretical models and biological complexity.

## Supporting information

S1 FigEffect of the growth rate and the parameters $\alpha_{R}$ and $\beta_{D}$ on pattern formation.Simulations were conducted on a growing mouse limb bud domain, as described in the main text. (**A**) is the growth rate from each mouse developmental stage with homogeneous and linearly growth rigidity. (**B**) shows the effect of the growth profile on the pattern with different values of $\alpha_{R}$ and $\beta_{D}$. (**C**) demonstrates the effect of removing the convective term. (**D**) represents the pattern without growth.(PDF)

S2 Fig Effect of the convective term on pattern formation in growing domains. **(A)** Simulation on a growing rectangular domain including the convective term, as described in the main text. **(B)** Simulation on a growing domain without the convective term. **(C)** Simulation on a static domain (no growth), where the convective term is absent by definition.
(PDF)

S3 FigEffect of growth rate distribution *S* on pattern formation in growing domains.**(A)** Simulation on a growing rectangular domain including the convective term with different distribution of growth rigidity across a range of $\alpha_{R}$ and $\beta_{D}$ values. **(B)** Similar simulations without the convective term.(PDF)

S4 FigEffect of the epithelium on pattern formation.(**A**) Pattern formation with an epithelium boundary condition. The epithelium is modeled by setting *u*(0) = 0 and *v*(0) = 0 in the first two layers of elements. The rest of the elements are initialized at the steady-state solution $\left(u_{0},v_{0}\right)$, defined as $𝐑(u_{0},v_{0})=0$. (**B**) Pattern formation without the epithelium. All elements are initialized at the steady-state solution $\left(u_{0},v_{0}\right)$.(PDF)

S5 FigEffect of the distribution of $\alpha_{R}$ and $\beta_{D}$ on the axolotl limb.The first column represents the framework without incorporating positional information (PI) or the apical ectodermal ridge (AER), implemented through homogeneous distributions of both $\alpha_{R}$ and $\beta_{D}$. The second column includes PI but excludes AER, modeled by a homogeneous $\beta_{D}$ and a spatially varying $\alpha_{R}$. The third column incorporates AER but excludes PI, implemented by a homogeneous $\alpha_{R}$ and a spatially varying $\beta_{D}$.(PDF)

S6 FigInfluence of $\beta_{D}$ distribution on limb bud patterning.(**A**) The intensity of $\beta_{D}$, (**B**) Change in AER thickness, and (**C**) the PD positioning of the AER influence patterning within the limb bud.(PDF)

S1 Table Parameter values used for different cases. (PDF)

S1 Text Turing space through linear stability analysis. This section presents the derivation of the equations governing the Growth-Reaction-Diffusion system and describes the linear stability analysis used to identify the range of parameters that ensure pattern formation.
(PDF)

## References

[1] Tickle C. The number of polarizing region cells required to specify additional digits in the developing chick wing. Nature. 1981;289(5795):295–8. doi: 10.1038/289295a0 7453825

[2] Shubin N, Tabin C, Carroll S. Deep homology and the origins of evolutionary novelty. Nature. 2009;457(7231):818–23. doi: 10.1038/nature07891 19212399

[3] Newman SA, Glimm T, Bhat R. The vertebrate limb: An evolving complex of self-organizing systems. Prog Biophys Mol Biol. 2018;137:12–24. doi: 10.1016/j.pbiomolbio.2018.01.002 29325895

[4] Saxena A, Towers M, Cooper KL. The origins, scaling and loss of tetrapod digits. Philos Trans R Soc Lond B Biol Sci. 2017;372(1713):20150482. doi: 10.1098/rstb.2015.0482 27994123 PMC5182414

[5] Stewart TA, Bhat R, Newman SA. The evolutionary origin of digit patterning. Evodevo. 2017;8:21. doi: 10.1186/s13227-017-0084-8 29201343 PMC5697439

[6] Wolpert L. Positional information and the spatial pattern of cellular differentiation. J Theor Biol. 1969;25(1):1–47. doi: 10.1016/s0022-5193(69)80016-0 4390734

[7] Johnson RL, Tabin CJ. Molecular models for vertebrate limb development. Cell. 1997;90(6):979–90. doi: 10.1016/s0092-8674(00)80364-5 9323126

[8] Mariani FV, Ahn CP, Martin GR. Genetic evidence that FGFs have an instructive role in limb proximal-distal patterning. Nature. 2008;453(7193):401–5. doi: 10.1038/nature06876 18449196 PMC2631409

[9] Zakany J, Duboule D. The role of Hox genes during vertebrate limb development. Curr Opin Genet Dev. 2007;17(4):359–66. doi: 10.1016/j.gde.2007.05.011 17644373

[10] Turing AM. The chemical basis of morphogenesis. 1953. Bull Math Biol. 1990;52(1–2):153–97; discussion 119-52. doi: 10.1007/BF02459572 2185858

[11] Green JBA, Sharpe J. Positional information and reaction-diffusion: two big ideas in developmental biology combine. Development. 2015;142(7):1203–11. doi: 10.1242/dev.114991 25804733

[12] Marcon L, Sharpe J. Turing patterns in development: what about the horse part?. Curr Opin Genet Dev. 2012;22(6):578–84. doi: 10.1016/j.gde.2012.11.013 23276682

[13] Sheth R, Marcon L, Bastida MF, Junco M, Quintana L, Dahn R, et al. Hox genes regulate digit patterning by controlling the wavelength of a Turing-type mechanism. Science. 2012;338(6113):1476–80. doi: 10.1126/science.1226804 23239739 PMC4486416

[14] Raspopovic J, Marcon L, Russo L, Sharpe J. Modeling digits. Digit patterning is controlled by a Bmp-Sox9-Wnt Turing network modulated by morphogen gradients. Science. 2014;345(6196):566–70. doi: 10.1126/science.1252960 25082703

[15] Grall E, Feregrino C, Fischer S, De Courten A, Sacher F, Hiscock TW, et al. Self-organized BMP signaling dynamics underlie the development and evolution of digit segmentation patterns in birds and mammals. Proc Natl Acad Sci U S A. 2024;121(2):e2304470121. doi: 10.1073/pnas.2304470121 38175868 PMC10786279

[16] Zeller R, López-Ríos J, Zuniga A. Vertebrate limb bud development: moving towards integrative analysis of organogenesis. Nat Rev Genet. 2009;10(12):845–58. doi: 10.1038/nrg2681 19920852

[17] Crampin EJ, Gaffney EA, Maini PK. Reaction and diffusion on growing domains: scenarios for robust pattern formation. Bull Math Biol. 1999;61(6):1093–120. doi: 10.1006/bulm.1999.0131 17879872

[18] Crampin EJ. Reaction-diffusion patterns on growing domains. University of Oxford. 2000.

[19] Madzvamuse A, Barreira R. Domain-growth-induced patterning for reaction-diffusion systems with linear cross-diffusion. Discrete and Continuous Dynamical Systems-B. 2018;23(7):2775–801.

[20] Krause AL, Ellis MA, Van Gorder RA. Influence of Curvature, Growth, and Anisotropy on the Evolution of Turing Patterns on Growing Manifolds. Bull Math Biol. 2019;81(3):759–99. doi: 10.1007/s11538-018-0535-y 30511207 PMC6373535

[21] Klika V, Gaffney EA. History dependence and the continuum approximation breakdown: the impact of domain growth on Turing’s instability. Proc Math Phys Eng Sci. 2017;473(2199):20160744. doi: 10.1098/rspa.2016.0744 28413340 PMC5378238

[22] Waters WA, Van Gorder RA. Turing patterns under hybrid apical–uniform domain growth. Proc R Soc A. 2025;481(2316). doi: 10.1098/rspa.2024.0935

[23] Krause AL, Gaffney EA, Walker BJ. Concentration-Dependent Domain Evolution in Reaction-Diffusion Systems. Bull Math Biol. 2023;85(2):14. doi: 10.1007/s11538-022-01115-2 36637542 PMC9839823

[24] Madzvamuse A, Gaffney EA, Maini PK. Stability analysis of non-autonomous reaction-diffusion systems: the effects of growing domains. J Math Biol. 2010;61(1):133–64. doi: 10.1007/s00285-009-0293-4 19727733

[25] Krause AL, Gaffney EA, Maini PK, Klika V. Modern perspectives on near-equilibrium analysis of Turing systems. Philos Trans A Math Phys Eng Sci. 2021;379(2213):20200268. doi: 10.1098/rsta.2020.0268 34743603 PMC8580451

[26] Fried P, Iber D. Dynamic scaling of morphogen gradients on growing domains. Nat Commun. 2014;5:5077. doi: 10.1038/ncomms6077 25295831

[27] Badugu A, Kraemer C, Germann P, Menshykau D, Iber D. Digit patterning during limb development as a result of the BMP-receptor interaction. Sci Rep. 2012;2:991. doi: 10.1038/srep00991 23251777 PMC3524521

[28] Miura T, Shiota K, Morriss-Kay G, Maini PK. Mixed-mode pattern in Doublefoot mutant mouse limb--Turing reaction-diffusion model on a growing domain during limb development. J Theor Biol. 2006;240(4):562–73. doi: 10.1016/j.jtbi.2005.10.016 16364368

[29] Hiscock TW, Tschopp P, Tabin CJ. On the Formation of Digits and Joints during Limb Development. Dev Cell. 2017;41(5):459–65. doi: 10.1016/j.devcel.2017.04.021 28586643 PMC5546220

[30] Uzkudun M, Marcon L, Sharpe J. Data-driven modelling of a gene regulatory network for cell fate decisions in the growing limb bud. Molecular systems biology. 2015;11(7):MSB145882. 10.15252/msb.20145882 PMC4547844 26174932

[31] Zhu J, Zhang Y-T, Alber MS, Newman SA. Bare bones pattern formation: a core regulatory network in varying geometries reproduces major features of vertebrate limb development and evolution. PLoS One. 2010;5(5):e10892. doi: 10.1371/journal.pone.0010892 20531940 PMC2878345

[32] Glimm T, Bhat R, Newman SA. Multiscale modeling of vertebrate limb development. Wiley Interdiscip Rev Syst Biol Med. 2020;12(4):e1485. doi: 10.1002/wsbm.1485 32212250

[33] Onimaru K, Marcon L, Musy M, Tanaka M, Sharpe J. The fin-to-limb transition as the re-organization of a Turing pattern. Nat Commun. 2016;7:11582. doi: 10.1038/ncomms11582 27211489 PMC4879262

[34] Nye HL, Cameron JA, Chernoff EA, Stocum DL. Extending the table of stages of normal development of the axolotl: limb development. Developmental dynamics: an official publication of the American Association of Anatomists. 2003;226(3):555–60. 10.1002/dvdy.10237 12619140

[35] Musy M, Flaherty K, Raspopovic J, Robert-Moreno A, Richtsmeier JT, Sharpe J. A quantitative method for staging mouse embryos based on limb morphometry. Development. 2018;145(7):dev154856. doi: 10.1242/dev.154856 29540505 PMC5963863

[36] Schindelin J, Arganda-Carreras I, Frise E, Kaynig V, Longair M, Pietzsch T, et al. Fiji: an open-source platform for biological-image analysis. Nat Methods. 2012;9(7):676–82. doi: 10.1038/nmeth.2019 22743772 PMC3855844

[37] Zienkiewicz OC, Taylor RL. The finite element method for solid and structural mechanics. Elsevier. 2005.

[38] Marcon L, Arqués CG, Torres MS, Sharpe J. A computational clonal analysis of the developing mouse limb bud. PLoS Comput Biol. 2011;7(2):e1001071. doi: 10.1371/journal.pcbi.1001071 21347315 PMC3037386

[39] Schnakenberg J. Simple chemical reaction systems with limit cycle behaviour. J Theor Biol. 1979;81(3):389–400. doi: 10.1016/0022-5193(79)90042-0 537379

[40] The MathWorks, Inc. MATLAB version R2023a. 2023.

[41] Lovely AM, Duerr TJ, Stein DF, Mun ET, Monaghan JR. Hybridization Chain Reaction Fluorescence In Situ Hybridization (HCR-FISH) in Ambystoma mexicanum Tissue. Methods Mol Biol. 2023;2562:109–22. doi: 10.1007/978-1-0716-2659-7_6 36272070 PMC10949069

[42] Woltering JM, Noordermeer D, Leleu M, Duboule D. Conservation and divergence of regulatory strategies at Hox Loci and the origin of tetrapod digits. PLoS Biol. 2014;12(1):e1001773. doi: 10.1371/journal.pbio.1001773 24465181 PMC3897358

[43] Sun X, Mariani FV, Martin GR. Functions of FGF signalling from the apical ectodermal ridge in limb development. Nature. 2002;418(6897):501–8. doi: 10.1038/nature00902 12152071

[44] Tabin C, Wolpert L. Rethinking the proximodistal axis of the vertebrate limb in the molecular era. Genes Dev. 2007;21(12):1433–42. doi: 10.1101/gad.1547407 17575045

[45] Summerbell D. Interaction between the proximo-distal and antero-posterior co-ordinates of positional value during the specification of positional information in the early development of the chick limb-bud. J Embryol Exp Morphol. 1974;32(1):227–37. doi: 10.1242/dev.32.1.227 4452831

[46] Zúñiga A, Haramis AP, McMahon AP, Zeller R. Signal relay by BMP antagonism controls the SHH/FGF4 feedback loop in vertebrate limb buds. Nature. 1999;401(6753):598–602. doi: 10.1038/44157 10524628

[47] Laufer E, Nelson CE, Johnson RL, Morgan BA, Tabin C. Sonic hedgehog and Fgf-4 act through a signaling cascade and feedback loop to integrate growth and patterning of the developing limb bud. Cell. 1994;79(6):993–1003. doi: 10.1016/0092-8674(94)90030-2 8001146

[48] Raines AM, Magella B, Adam M, Potter SS. Key pathways regulated by HoxA9, 10, 11/HoxD9, 10, 11 during limb development. BMC Developmental Biology. 2015;15:1–15. 26186931 10.1186/s12861-015-0078-5 PMC4506574

[49] Mao J, McGlinn E, Huang P, Tabin CJ, McMahon AP. Fgf-dependent Etv4/5 activity is required for posterior restriction of Sonic Hedgehog and promoting outgrowth of the vertebrate limb. Dev Cell. 2009;16(4):600–6. doi: 10.1016/j.devcel.2009.02.005 19386268 PMC3164484

[50] Zhang Z, Verheyden JM, Hassell JA, Sun X. FGF-regulated Etv genes are essential for repressing Shh expression in mouse limb buds. Dev Cell. 2009;16(4):607–13. doi: 10.1016/j.devcel.2009.02.008 19386269 PMC3541528

[51] Fröbisch NB, Shubin NH. Salamander limb development: integrating genes, morphology, and fossils. Dev Dyn. 2011;240(5):1087–99. doi: 10.1002/dvdy.22629 21465623

[52] Gardiner DM, Blumberg B, Komine Y, Bryant SV. Regulation of HoxA expression in developing and regenerating axolotl limbs. Development. 1995;121(6):1731–41. doi: 10.1242/dev.121.6.1731 7600989

[53] Torok MA, Gardiner DM, Izpisúa-Belmonte JC, Bryant SV. Sonic hedgehog (shh) expression in developing and regenerating axolotl limbs. J Exp Zool. 1999;284(2):197–206. doi: 10.1002/(sici)1097-010x(19990701)284:2<197::aid-jez9>3.3.co;2-6 10404648

[54] Zhong J, Aires R, Tsissios G, Skoufa E, Brandt K, Sandoval-Guzmán T, et al. Multi-species atlas resolves an axolotl limb development and regeneration paradox. Nat Commun. 2023;14(1):6346. doi: 10.1038/s41467-023-41944-w 37816738 PMC10564727

[55] Nacu E, Gromberg E, Oliveira CR, Drechsel D, Tanaka EM. FGF8 and SHH substitute for anterior-posterior tissue interactions to induce limb regeneration. Nature. 2016;533(7603):407–10. doi: 10.1038/nature17972 27120163

[56] Glotzer GL, Tardivo P, Tanaka EM. Canonical Wnt signaling and the regulation of divergent mesenchymal Fgf8 expression in axolotl limb development and regeneration. Elife. 2022;11:e79762. doi: 10.7554/eLife.79762 35587651 PMC9154742

[57] Purushothaman S, Elewa A, Seifert AW. Fgf-signaling is compartmentalized within the mesenchyme and controls proliferation during salamander limb development. Elife. 2019;8:e48507. doi: 10.7554/eLife.48507 31538936 PMC6754229

[58] Lovely AM, Duerr TJ, Qiu Q, Galvan S, Voss SR, Monaghan JR. Wnt Signaling Coordinates the Expression of Limb Patterning Genes During Axolotl Forelimb Development and Regeneration. Front Cell Dev Biol. 2022;10:814250. doi: 10.3389/fcell.2022.814250 35531102 PMC9068880

[59] Chen Y, Zhao X. Shaping limbs by apoptosis. J Exp Zool. 1998;282(6):691–702. doi: 10.1002/(sici)1097-010x(19981215)282:6<691::aid-jez5>3.0.co;2-s 9846381

[60] Roensch K, Tazaki A, Chara O, Tanaka EM. Progressive specification rather than intercalation of segments during limb regeneration. Science. 2013;342(6164):1375–9. doi: 10.1126/science.1241796 24337297

[61] Witte F, Dokas J, Neuendorf F, Mundlos S, Stricker S. Comprehensive expression analysis of all Wnt genes and their major secreted antagonists during mouse limb development and cartilage differentiation. Gene Expr Patterns. 2009;9(4):215–23. doi: 10.1016/j.gep.2008.12.009 19185060

[62] Mosby LS, Bowen AE, Hadjivasiliou Z. Morphogens in the evolution of size, shape and patterning. Development. 2024;151(18):dev202412. doi: 10.1242/dev.202412 39302048 PMC7616732

[63] Kondo S, Miura T. Reaction-diffusion model as a framework for understanding biological pattern formation. Science. 2010;329(5999):1616–20. doi: 10.1126/science.1179047 20929839

[64] Murray JD. Spatial models and biomedical applications. Mathematical Biology. 2003.

[65] Goriely A. The mathematics and mechanics of biological growth. Springer. 2017.

[66] Boehm B, Westerberg H, Lesnicar-Pucko G, Raja S, Rautschka M, Cotterell J, et al. The role of spatially controlled cell proliferation in limb bud morphogenesis. PLoS Biol. 2010;8(7):e1000420. doi: 10.1371/journal.pbio.1000420 20644711 PMC2903592

[67] Morishita Y, Suzuki T. Bayesian inference of whole-organ deformation dynamics from limited space-time point data. J Theor Biol. 2014;357:74–85. doi: 10.1016/j.jtbi.2014.04.027 24810841

[68] Lesnicar-Pucko G, Belmonte JM, Musy M, Glazier JA, Sharpe J. Cellular mechanisms of chick limb bud morphogenesis. BioRxiv. 2020;:2020–09.

[69] ten Berge D, Brugmann SA, Helms JA, Nusse R. Wnt and FGF signals interact to coordinate growth with cell fate specification during limb development. Development. 2008;135(19):3247–57. doi: 10.1242/dev.023176 18776145 PMC2756806

[70] Aviñó-Esteban L, Cardona-Blaya H, Sharpe J. Spatio-temporal reconstruction of gene expression patterns in developing mice. Development. 2025;152(4):DEV204313. doi: 10.1242/dev.204313 39982400 PMC11883288

[71] Dillon R, Othmer HG. A mathematical model for outgrowth and spatial patterning of the vertebrate limb bud. J Theor Biol. 1999;197(3):295–330. doi: 10.1006/jtbi.1998.0876 10089144

[72] Sears KE, Behringer RR, Rasweiler JJ 4th, Niswander LA. Development of bat flight: morphologic and molecular evolution of bat wing digits. Proc Natl Acad Sci U S A. 2006;103(17):6581–6. doi: 10.1073/pnas.0509716103 16618938 PMC1458926

[73] Darnet S, Dragalzew AC, Amaral DB, Sousa JF, Thompson AW, Cass AN, et al. Deep evolutionary origin of limb and fin regeneration. Proc Natl Acad Sci U S A. 2019;116(30):15106–15. doi: 10.1073/pnas.1900475116 31270239 PMC6660751

