# Supplementary material for: Development of whole-limb skeletal patterning through the coordination of growth and self-organization models
Source: PLoS Comput Biol. 2026 Jul 7;22(7):e1014348. doi: 10.1371/journal.pcbi.1014348 (PMC13384404; doi:10.1371/journal.pcbi.1014348)
Supplement: S1 Fig — Simulations were conducted on a growing mouse limb bud domain, as described in the main text. (A) is the growth rate from each mouse developmental stage with homogeneous and linearly growth rigidity. (B) shows the effect of the growth profile on the pattern with different values of αR and βD. (C) demonstrates the effect of removing the convective term. (D) represents the pattern without growth. (PDF) [file pcbi.1014348.s001.pdf]

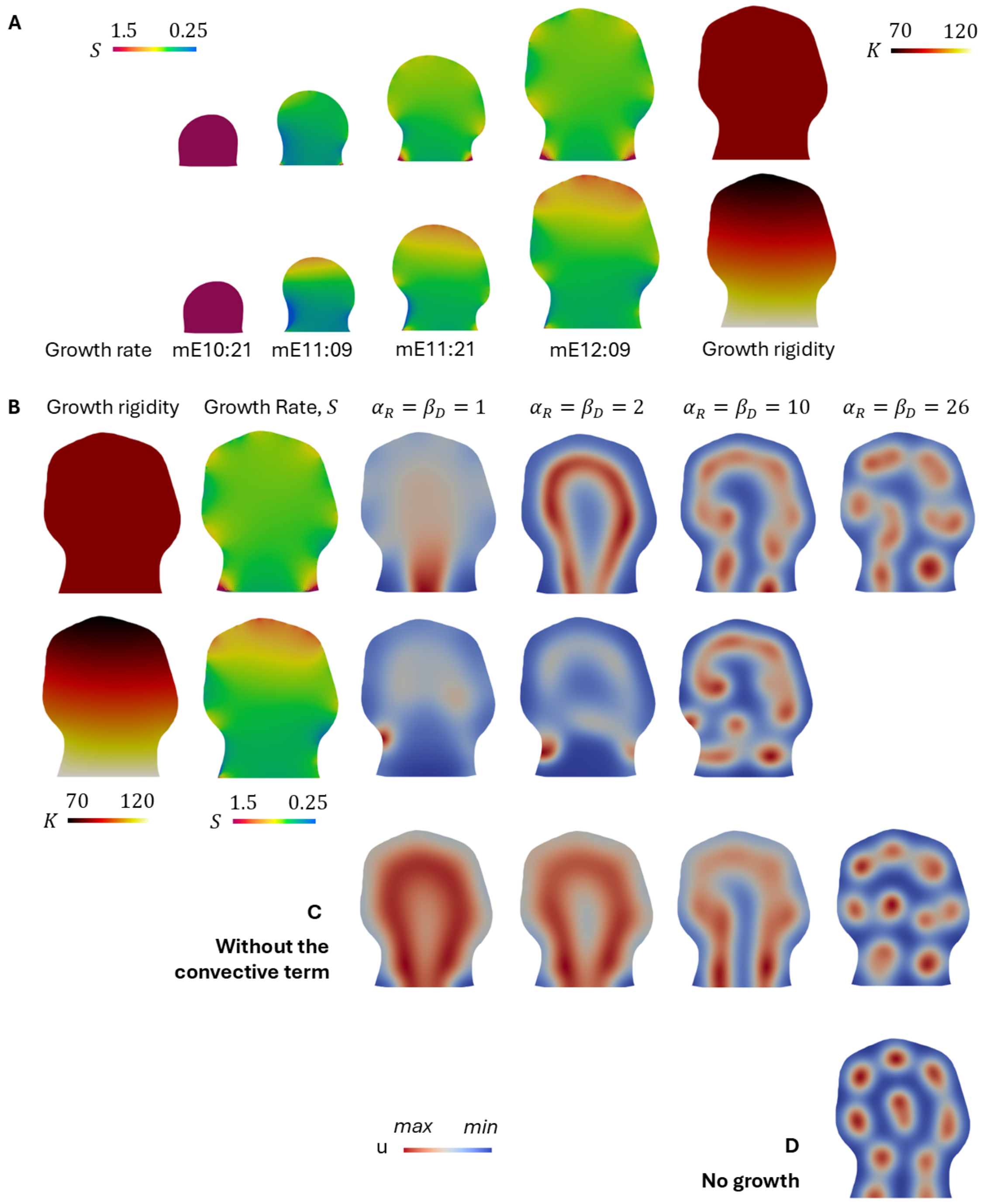

Figure S1. Effect of the growth rate and the parameters α_R and β_D on pattern formation. Simulations were conducted on a growing mouse limb bud domain, as described in the main text. All simulations were run with α_R and β_D kept constant and homogeneous throughout the growth process.

A. Growth profile throughout the limb development. The first row shows the growth rate from each developmental stage with homogeneous growth rigidity, while the second row shows a linearly varying growth rigidity. **B. Effect of the growth profile on the pattern.** The first row shows results with homogeneous growth rigidity, while the second row shows a linearly varying growth rigidity, producing distinct growth patterns. The top-left panel (convective term, $\alpha_R = \beta_D = 1$) corresponds to Fig.2A1 in the main text. At low values of α_R and β_D, the spatial distribution of growth strongly affects the final pattern. At high values, reaction-diffusion dynamics dominate, producing patterns similar to those obtained without growth (shown in **C**).

C. Effect of removing the convective term. The third row shows patterns generated without the convective term. Even without convection, high values of α_R and β_D produce patterns comparable to the no growth simulations.

D. Pattern without growth. The reaction-diffusion simulation ran directly on the final limb bud geometry. Here, the values of α_R and β_D do not affect the pattern, as shown in Fig.S2.

These observations are consistent with what was shown on the growing rectangle in the main text (Fig.6).
