# Supplementary material for: Development of whole-limb skeletal patterning through the coordination of growth and self-organization models
Source: PLoS Comput Biol. 2026 Jul 7;22(7):e1014348. doi: 10.1371/journal.pcbi.1014348 (PMC13384404; doi:10.1371/journal.pcbi.1014348)
Supplement: S2 Fig — (A) Simulation on a growing rectangular domain including the convective term, as described in the main text. (B) Simulation on a growing domain without the convective term. (C) Simulation on a static domain (no growth), where the convective term is absent by definition. (PDF) [file pcbi.1014348.s002.pdf]

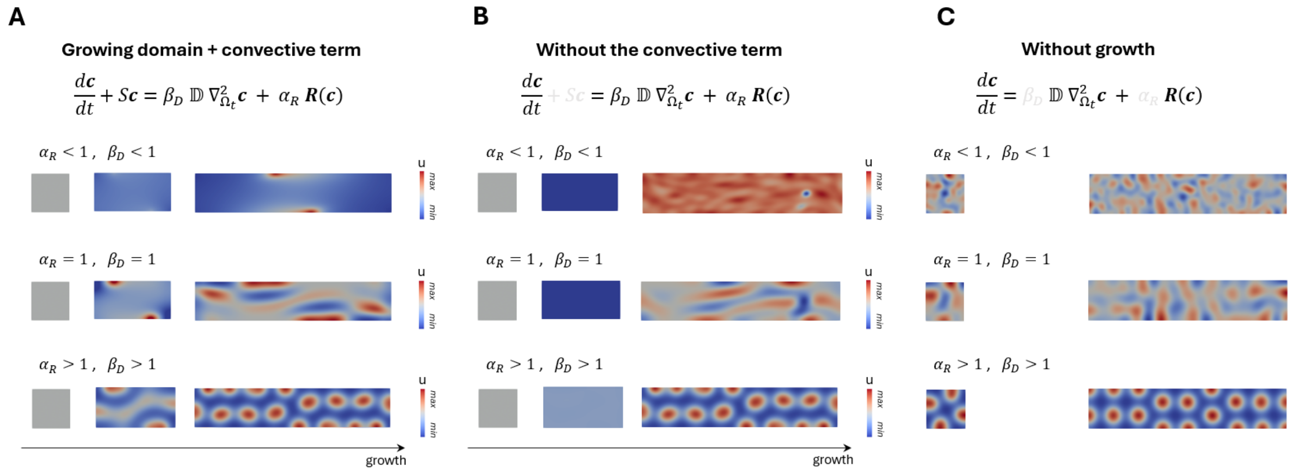

Figure S2. Effect of the convective term on pattern formation in growing domains. (A) Simulation on a growing rectangular domain including the convective term, as described in the main text. **(B)** Simulation on a growing domain without the convective term. Lower values of α_R and β_D show a noticeable impact of the convective term on the resulting pattern. **(C)** Simulation on a static domain (no growth), where the convective term is absent by definition. In this case, α_R and β_D lose their influence on the pattern's shape, and all simulations yield the same final pattern. Higher values of α_R and β_D only accelerate the convergence of the pattern.
