# Supplementary material for: Development of whole-limb skeletal patterning through the coordination of growth and self-organization models
Source: PLoS Comput Biol. 2026 Jul 7;22(7):e1014348. doi: 10.1371/journal.pcbi.1014348 (PMC13384404; doi:10.1371/journal.pcbi.1014348)
Supplement: S3 Fig — (A) Simulation on a growing rectangular domain including the convective term with different distribution of growth rigidity across a range of αR and βD values. (B) Similar simulations without the convective term. (PDF) [file pcbi.1014348.s003.pdf]

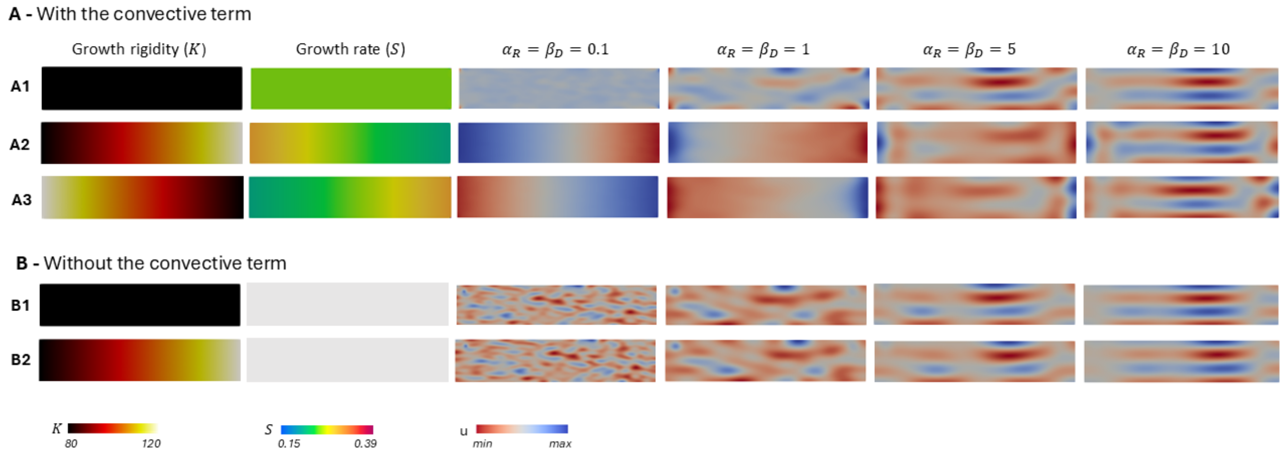

Figure S3. Effect of growth rate distribution S on pattern formation in growing domains. (A) Simulations on a growing rectangular domain including the convective term, as described in the main text. Each line corresponds to a different distribution of growth rigidity: A1 represents homogeneous rigidity, resulting in a uniform growth rate; A2 and A3 show a linear variation in rigidity, producing a linear gradient in growth rate. Each simulation was run across a range of α_R and β_D values. At low values of α_R and β_D, the spatial distribution of growth significantly affects pattern formation. In contrast, at high values of these parameters, reactionâdiffusion dynamics dominate, leading to patterns that resemble those obtained in the absence of growth. **(B)** To assess whether pattern transport is a numerical artifact of the evolving mesh, simulations were repeated without the convective term, using homogeneous (B1) and linear (B2) growth rigidity profiles. The fact that B1 and B2 yield identical results confirms that the patterns are independent of mesh deformation. Moreover, the results show that, even without the convective term, high α_R and β_D values lead to patterns comparable to those obtained with growth-free simulations, confirming the dominance of RD dynamics under these conditions.
