# Supplementary material for: Development of whole-limb skeletal patterning through the coordination of growth and self-organization models
Source: PLoS Comput Biol. 2026 Jul 7;22(7):e1014348. doi: 10.1371/journal.pcbi.1014348 (PMC13384404; doi:10.1371/journal.pcbi.1014348)
Supplement: S4 Fig — (A) Pattern formation with an epithelium boundary condition. The epithelium is modeled by setting u(0) = 0 and v(0) = 0 in the first two layers of elements. The rest of the elements are initialized at the steady-state solution (u0,v0), defined as 𝐑(u0,v0)=0. (B) Pattern formation without the epithelium. All elements are initialized at the steady-state solution (u0,v0). (PDF) [file pcbi.1014348.s004.pdf]

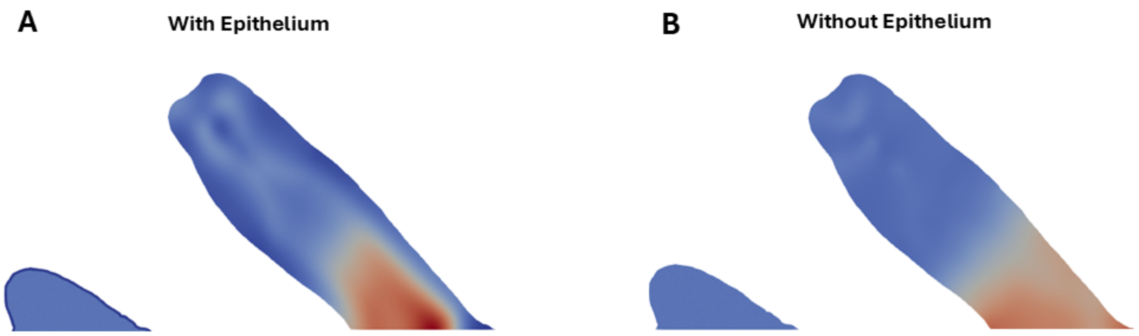

Figure S4. Effect of the epithelium on pattern formation. (**A**) Pattern formation with an epithelium boundary condition. The epithelium is modeled by setting $u(0) = 0$ and $v(0) = 0$ in the first two layers of mesh elements. The rest of the elements are initialized at the steady-state solution (u_0, v_0), defined as $\mathbf{R}(u_0, v_0) = \mathbf{0}$. (**B**) Pattern formation without the epithelium. All elements are initialized at a random 10% value around the steady-state solution (u_0, v_0). Both cases have different initial conditions at $t = 0$, without any additional specification on the epithelium for $t > 0$.
