# Supplementary material for: Development of whole-limb skeletal patterning through the coordination of growth and self-organization models
Source: PLoS Comput Biol. 2026 Jul 7;22(7):e1014348. doi: 10.1371/journal.pcbi.1014348 (PMC13384404; doi:10.1371/journal.pcbi.1014348)
Supplement: S5 Fig — The first column represents the framework without incorporating positional information (PI) or the apical ectodermal ridge (AER), implemented through homogeneous distributions of both αR and βD. The second column includes PI but excludes AER, modeled by a homogeneous βD and a spatially varying αR. The third column incorporates AER but excludes PI, implemented by a homogeneous αR and a spatially varying βD. (PDF) [file pcbi.1014348.s005.pdf]

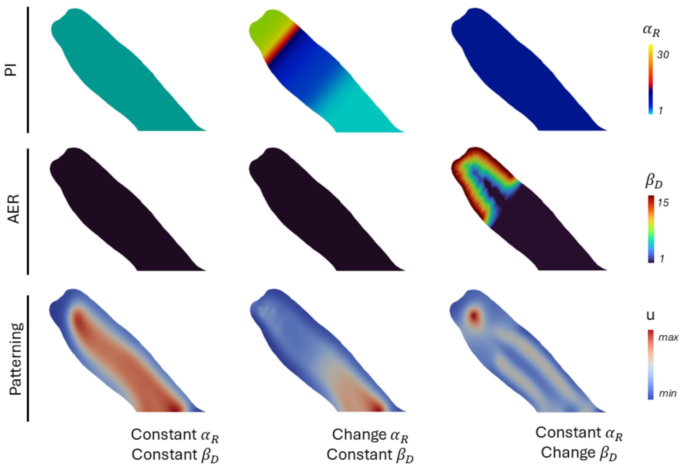

Figure S5. Effect of the distribution of α_R and β_D on the axolotl limb. The first column represents the framework without incorporating positional information (PI) or the apical ectodermal ridge (AER), implemented through homogeneous and constant distributions of both α_R and β_D, resulting in a simple stripe pattern. The second column includes PI but excludes AER, modeled by a homogeneous β_D and a spatially varying α_R, maintaining the proximal pattern while losing autopod features. The third column incorporates AER but excludes PI, implemented by a homogeneous α_R and a spatially varying β_D, where PI takes a uniform value corresponding to the zeugopod. As a result, the model produces an extended zeugopod-like element. This figure shows the axolotl counterpart of the results presented in Fig.2A.
