# Supplementary material for: Development of whole-limb skeletal patterning through the coordination of growth and self-organization models
Source: PLoS Comput Biol. 2026 Jul 7;22(7):e1014348. doi: 10.1371/journal.pcbi.1014348 (PMC13384404; doi:10.1371/journal.pcbi.1014348)
Supplement: S6 Fig — (A) The intensity of βD, (B) Change in AER thickness, and (C) the PD positioning of the AER influence patterning within the limb bud. (PDF) [file pcbi.1014348.s006.pdf]

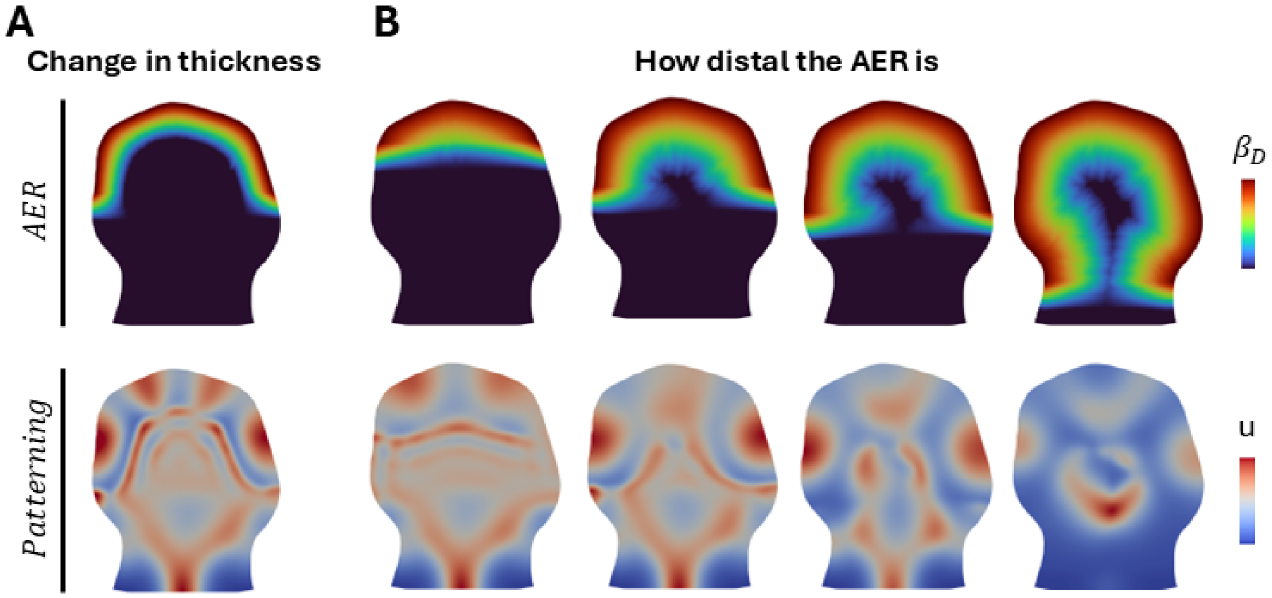

Figure S6. Influence of β_D distribution on limb bud patterning. (**A**) Change in AER thickness, and (**B**) the proximodistal positioning of the AER influence patterning within the limb bud. The top row shows the resulting patterning in the limb, while the bottom row shows the corresponding AER. Color scales indicate the spatial variations in AER properties through β_D distribution (top row) and patterning (bottom row, u).
