# Supplementary material for: Development of whole-limb skeletal patterning through the coordination of growth and self-organization models
Source: PLoS Comput Biol. 2026 Jul 7;22(7):e1014348. doi: 10.1371/journal.pcbi.1014348 (PMC13384404; doi:10.1371/journal.pcbi.1014348)
Supplement: S1 Table — (PDF) [file pcbi.1014348.s007.pdf]

Table S1. Parameter values used for different cases. The parameters k_1 and k_2 correspond to the kinetic terms of the Schnakenberg system, as described in Eq. (11). The relative diffusion of v with respect to u (d) is seen in Eq. (S.10). The parameters α_R, α_{R_1}, and α_{R_2} represent the values of positional information shown in Fig. 1B and Fig. 3B. The parameters l_1 and l_2 define the transition positions between the stylopod and zeugopod, and between the zeugopod and autopod regions, respectively, relative to the final limb length. The parameters a_1 and a_2 control the smoothness of these transitions. The parameters β_D and β_{D_1} indicate the AER values shown in Fig.1B and Fig.3F. Finally, N represents the number of elements used to define the thickness of the AER. AER parameters are explored in SUPP. S6.

	**Turing Space**			α_R							β_D		
Parameters	k_1	k_2	d	α_R	α_{R_1}	l_1	a_1	α_{R_2}	l_2	a_2	β_D	β_{D_1}	N
Mice	0.47	2	113	1	2	0.13	0.03	8	0.45	0.03	1	26	30
Axolotl	0.47	2	113	1	5	0.25	0.05	35	0.75	0.05	1	15	12
