# Supplementary material for: Development of whole-limb skeletal patterning through the coordination of growth and self-organization models
Source: PLoS Comput Biol. 2026 Jul 7;22(7):e1014348. doi: 10.1371/journal.pcbi.1014348 (PMC13384404; doi:10.1371/journal.pcbi.1014348)
Supplement: S1 Text — This section presents the derivation of the equations governing the Growth-Reaction-Diffusion system and describes the linear stability analysis used to identify the range of parameters that ensure pattern formation. (PDF) [file pcbi.1014348.s008.pdf]

S1 Turing space through linear stability analysis

This section presents the derivation of the equations governing the Growth-Reaction-Diffusion system and describes the linear stability analysis used to identify the range of parameters that ensure pattern formation.

Derivation of the reaction-diffusion equation in a growing domain

We consider a growing domain Ω_x enclosed by a surface $\partial\Omega_x$. From the general conservation equation, the amount of material in Ω_x is equal to the rate of growth velocity of this material across $\partial\Omega_x$ into Ω_x, plus the material created in Ω_x. For a species ξ with a concentration c, this can be expressed by

$$\frac{d}{dt}\int_{\Omega_x} c(\mathbf{x},t)d\mathbf{x} = -\int_{\partial\Omega_x} \mathbf{j}\cdot\mathbf{ds} + \int_{\Omega_x} R\,d\mathbf{x}, \tag{1}$$

where $\mathbf{j}$ is the flux of material going through $\mathbf{ds} = \mathbf{n}\,ds$ with $\mathbf{n}$ the normal of the surface element ds. The source of material is R, which can depend on the vector of species concentration c, the position vector $\mathbf{x}$, and the time t. By applying the divergence theorem to the surface integral, the previous equation becomes

$$\frac{d}{dt}\int_{\Omega_x} c(\mathbf{x},t)d\mathbf{x} = \int_{\Omega_x} \left[-\nabla\cdot\mathbf{j} + R(c,\mathbf{x},t)\right]d\mathbf{x}. \tag{2}$$

In order to evaluate the left-hand term, we use the Reynolds transport theorem

$$\frac{d}{dt}\int_{\Omega_x} c(\mathbf{x},t)d\mathbf{x} = \int_{\Omega_x} \left[\frac{\partial c}{\partial t} + \nabla\cdot(\mathbf{a}c)\right]d\mathbf{x}, \tag{3}$$

where $\mathbf{a}(\mathbf{x},t)$ is the growth velocity, and the evolution equation becomes

$$\int_{\Omega_x} \left[\frac{\partial c}{\partial t} + \nabla\cdot\mathbf{a}c\right]d\mathbf{x} = \int_{\Omega_x} \left[-\nabla\cdot\mathbf{j} + R(c,\mathbf{x},t)\right]d\mathbf{x}. \tag{4}$$

As this must hold true for any t, the integrands must be equal to each other. Therefore, we obtain the *local conservation equation* for c which is

$$\frac{\partial c}{\partial t} + \nabla_{\Omega_x}\cdot(\mathbf{a}c) = -\nabla_{\Omega_x}\cdot\mathbf{j} + R(c,\mathbf{x},t). \tag{5}$$

Here, $\mathbf{j}$ is a general flux transport vector, which can be diffusion or another process. We consider a classical diffusion problem,

$$\mathbf{j} = -D\nabla_{\Omega_x} c, \tag{6}$$

where D is the diffusivity at constant density. Equation (5) becomes

$$\frac{\partial c}{\partial t} + \nabla_{\Omega_x}\cdot(\mathbf{a}c) = \nabla_{\Omega_x}\cdot(D\nabla_{\Omega_x} c) + R(c,\mathbf{x},t), \tag{7}$$

where D may be a function of $\mathbf{x}$ and c. The time-varying domain introduces two terms: $\nabla c\cdot\mathbf{a}$ and $c\,\nabla\cdot\mathbf{a}$. The first represents the transport of material around the domain at a certain rate (dependent of the growth velocity $\mathbf{a}$), in other terms it corresponds to the elemental volume moving with the growth velocity. The second one represents the dilution effect due to the local volume increase. The equation becomes

$$\frac{\partial c}{\partial t} + \nabla_{\Omega_x} c\cdot\mathbf{a} + c\,\nabla_{\Omega_x}\cdot\mathbf{a} = D\nabla^2_{\Omega_x} c + R(c,\mathbf{x},t), \tag{8}$$

where D has been assumed to be independent of the spatial variable $\mathbf{x}$. The final equation is

$$\frac{dc}{dt} + S(\mathbf{x},t)\,c = D\nabla^2_{\Omega_x} c + R(c,\mathbf{x},t), \tag{9}$$

where $S(\mathbf{x},t)$ is the growth rate.

Linear stability analysis in a growing domain

We perform a linear stability analysis to determine the Turing space of parameters in a growing domain. First, we deduce the form of growth that yields a constant growth rate S in the linearized system. Then, we analyze its stability both with and without diffusion (1). For pattern formation to occur, the system must be stable without diffusion and unstable when diffusion is present. These conditions yield four equations that define the set of parameters capable of generating patterns.

The linear stability analysis is then performed on a two-species system with $\mathbf{c} = (u, v)$,

$$\begin{aligned}\frac{du}{dt} &= \beta_D \nabla^2_{\Omega_x} u + \alpha_R f(u, v) - uS(\mathbf{x}, t), \\ \frac{dv}{dt} &= \beta_D d \nabla^2_{\Omega_x} v + \alpha_R g(u, v) - vS(\mathbf{x}, t),\end{aligned} \tag{10}$$

where $f(u, v)$ and $g(u, v)$ are the reaction terms for species u and v, respectively. The form of the functions follow the Schnakenberg kinetics (2)

$$\begin{aligned}f(u, v) &= a - u + u^2 v, \\ g(u, v) &= b - u^2 v,\end{aligned} \tag{11}$$

where a and b are model parameters. The ratio of their diffusivities is given by $d = D_v/D_u$. The parameters α_R and β_D are non-dimensional quantities. Our objective is to identify a homogeneous steady state that remains stable under small perturbations in the absence of diffusion, but becomes unstable when small spatial perturbations are introduced in the presence of diffusion.

The homogeneous steady state is defined as the solution of the system in (10) with both time and space independence. This solution can only be found if we assume that $S(\mathbf{x}, t)$ is a constant S. The homogeneous steady state can be determined by solving

$$\alpha_R f(u_0, v_0) - u_0 S = 0 \quad \text{and} \quad \alpha_R g(u_0, v_0) - v_0 S = 0. \tag{12}$$

Introducing a small perturbation to the homogeneous steady state,

$$\begin{aligned}u &= u_0 + w_1(\mathbf{x}, t), \\ v &= v_0 + w_2(\mathbf{x}, t),\end{aligned} \tag{13}$$

and substituting it into (10), the linearized system

$$\begin{bmatrix} w_{1t} \\ w_{2t} \end{bmatrix} = \beta_D \begin{bmatrix} 1 & 0 \\ 0 & d \end{bmatrix} \nabla^2_{\Omega_x} \begin{bmatrix} w_1 \\ w_2 \end{bmatrix} + \alpha_R \begin{bmatrix} f_u & f_v \\ g_u & g_v \end{bmatrix}_{(u_0, v_0)} \begin{bmatrix} w_1 \\ w_2 \end{bmatrix} - S \begin{bmatrix} w_1 \\ w_2 \end{bmatrix} \tag{14}$$

is obtained, where the subindex t indicates a time derivative, $\mathbb{A}$ is the Jacobian matrix of f and g evaluated at (u_0, v_0),

$$\mathbb{A} = \begin{bmatrix} f_u & f_v \\ g_u & g_v \end{bmatrix}_{(u_0, v_0)}, \tag{15}$$

and

$$\mathbb{D} = \begin{bmatrix} 1 & 0 \\ 0 & d \end{bmatrix}, \tag{16}$$

is the diffusivity matrix.

Growth function for the linear analysis

To determine the linearized solution, the growth rate $S(\mathbf{x}, t)$ must be constant in both time and space. The goal is to identify a growth function $\mathbf{x}(t) = \mathbf{\Gamma}(\mathbf{X}, t)$ that yields a constant S. For simplicity, the analysis is restricted to one dimension.

An exponential growth function is first considered,

$$x = X\,\mathrm{e}^{\kappa t}, \tag{17}$$

with growth velocity

$$a = \frac{\partial \Gamma}{\partial t} = \kappa X\,\mathrm{e}^{\kappa t} = \kappa x, \tag{18}$$

leading to a constant growth rate

$$S = \frac{\partial a}{\partial x} = \kappa. \tag{19}$$

This exponential form maintains a constant $S(x, t)$, satisfying the requirements for the linear stability analysis.

For comparison, a linear growth function is also examined,

$$x = X + X\kappa t \Longrightarrow X = \frac{x}{1+\kappa t}, \tag{20}$$

where κ is a known scalar. The corresponding growth velocity is

$$a(x,t) = \frac{\partial \Gamma}{\partial t} = \kappa X = \kappa\,\frac{x}{1+\kappa t}, \tag{21}$$

yielding a time-dependent growth rate,

$$S(x,t) = \frac{\partial a}{\partial x} = \frac{\kappa}{1+\kappa t}. \tag{22}$$

In this case, $S(\mathbf{x}, t)$ is unsuitable for the linearization. In the axolotl limb, experimental measurements suggest that growth is approximately linear in time, though spatially non-uniform. Therefore, the assumption of a constant S made for this analysis does not appear to be supported by experimental evidence. However, we proceeded with this simplification in order to obtain guidelines for identifying the Turing space of parameters for the model parameters.

Linearized solution

We assume a general form for the solution to (10)

$$\begin{bmatrix} w_1(\mathbf{x},t) \\ w_2(\mathbf{x},t) \end{bmatrix} = \exp(\lambda t) \begin{bmatrix} \Phi_1(\mathbf{x}) \\ \Phi_2(\mathbf{x}) \end{bmatrix}, \tag{23}$$

where λ is an Eigenvalue of $\alpha_R \mathbb{A}$, and $\Phi_{1,2}(\mathbf{x})$ are the components of the Eigenvector of the spatial problem. Substituting this into the linearized equation (14) yields

$$\beta_D \mathbb{D} \nabla^2 \mathbf{\Phi} + [\alpha_R \mathbb{A} - [\lambda + S]\,\mathbb{I}\,]\,\mathbf{\Phi} = \mathbf{0}, \tag{24}$$

where $\mathbb{I}$ is the 2×2 identity matrix and $\mathbf{\Phi}$ is the vector $[\Phi_1, \Phi_2]$. The solution that satisfies (24) can be expressed as $\mathbf{\Phi}_m = \mathbf{y}_m \Phi_m$, where $\mathbf{y}_m$ is a constant vector and $\Phi_m(\mathbf{x})$ is a scalar Eigenfunction of the Laplacian satisfying the boundary conditions. This implies that $\nabla^2 \Phi_m(\mathbf{x}) = -k_m^2 \Phi_m(\mathbf{x})$, where k_m is the wavenumber corresponding to each Φ_m. Therefore, (24) becomes

$$[-\beta_D \mathbb{D} k_m^2 + [\alpha_R \mathbb{A} - [\lambda + S]\,\mathbb{I}\,]\,\mathbf{y}_m = \mathbf{0}. \tag{25}$$

For non-trivial solutions, the determinant must satisfy

$$| - \beta_D \mathbb{D} k_m^2 + [\alpha_R \mathbb{A} - [\lambda + S]\,\mathbb{I}\,]| = 0, \tag{26}$$

leading to the dispersion relation $\lambda = \lambda(k_m^2)$. It can be written as

$$\lambda^2 + \lambda\left[\beta_D\,[1+d]\,k_m^2 - \alpha_R\,[f_u + g_v] + 2\,S\right] + h(k_m^2) = 0, \tag{27}$$

where

$$h(k_m^2) = k_m^4\,\beta_D^2 d + k_m^2\,\beta_D\Big[[1+d]\,S - \alpha_R\,[d\,f_u + g_v]\Big] + \Big[\alpha_R^2[f_u\,g_v - f_v\,g_u] - \alpha_R\,[f_u + g_v]\,S + S^2\Big]. \tag{28}$$

Stability without diffusion

To determine the stability of the solution, we examine the sign of the real part of $\lambda(k_m^2)$. If $\mathfrak{Re}(\lambda) < 0$, the solution is stable (decreasing in time), and if $\mathfrak{Re}(\lambda) > 0$, the solution is unstable (increasing exponentially in time). Without diffusion ($k = 0$), the equation becomes

$$\lambda^2 - \lambda\,[\,\alpha_R\,[f_u + g_v] - 2\,S\,] + \alpha_R^2\,[f_u\,g_v - f_v\,g_u] - \alpha_R\,[f_u + g_v]\,S + S^2 = 0, \tag{29}$$

whose roots are

$$2\lambda_0^{\pm} = [\,\alpha_R\,[f_u + g_v] - 2\,S] \pm \sqrt{(\,\alpha_R\,[f_u + g_v] - 2\,S)^2 - 4[\alpha_R^2\,[f_u\,g_v - f_v\,g_u] - \alpha_R\,[f_u + g_v]\,S + S^2]}. \tag{30}$$

For λ_0^-, the condition for stability is

$$\alpha_R\,[f_u + g_v] - 2\,S < 0, \tag{31}$$

which can be rewritten in terms of the trace of $\mathbb{A}$ to obtain the first condition

$$\alpha_R\,tr(\mathbb{A}) - 2S < 0. \tag{32}$$

For λ_0^+ to be negative, we require

$$[\,\alpha_R\,[f_u + g_v] - 2\,S] > \sqrt{(\,\alpha_R\,[f_u + g_v] - 2\,S)^2 - 4[\alpha_R^2\,[f_u\,g_v - f_v\,g_u] - \alpha_R\,[f_u + g_v]\,S + S^2]}, \tag{33}$$

which can be written as

$$\alpha_R^2\,[f_u\,g_v - f_v\,g_u] - \alpha_R\,[f_u + g_v]\,S + S^2 > 0, \tag{34}$$

resulting in

$$\alpha_R^2\,|\mathbb{A}| - \alpha_R\,tr(\mathbb{A})\,S + S^2 > 0, \tag{35}$$

which is the second condition.

Instability with diffusion

When considering diffusion in equation (27), the roots are

$$2\,\lambda^{\pm}(k_m^2) = -\left[\beta_D\,[1 + d]\,k_m^2 - \alpha_R\,[f_u + g_v] + 2\,S\right] \pm \sqrt{\left(\beta_D\,[1 + d]\,k_m^2 - \alpha_R\,[f_u + g_v] + 2\,S\right)^2 - 4h(k_m^2)}, \tag{36}$$

Given the condition for stability (32), necessarily

$$\beta_D\,[1 + d]\,k_m^2 - \alpha_R\,[f_u + g_v] + 2\,S > 0, \tag{37}$$

and therefore, $\lambda^-(k_m^2)$ is always negative. To ensure instability, $\lambda^+(k_m^2)$ must be positive, which requires

$$h(k_m^2) < 0.$$

This leads to the condition

$$k_m^4\,\beta_D^2 d + k_m^2\,[\,[1 + d]\,S - \alpha_R\,[d\,f_u + g_v]]\,\beta_D\, + \left[\alpha_R^2[f_u\,g_v - f_v\,g_u] - \alpha_R\,[f_u + g_v]\,S + S^2\right] < 0, \tag{38}$$

which, given the condition for stability (35), can be simplified to

$$[1 + d]\,S - \alpha_R\,[d\,f_u + g_v] < 0. \tag{39}$$

This relation provides a necessary condition but is not sufficient on its own. To establish a condition that is both necessary and sufficient, the minimum value $h(k_m^2)$ must be negative. This leads to the following condition

$$\frac{1}{4d}\left(\,[1 + d]\,S - \alpha_R\,[d\,f_u + g_v]\right)^2 - \left[\alpha_R^2|\mathbb{A}| - \alpha_R\,tr(\mathbb{A})\,S + S^2\right] > 0. \tag{40}$$

Instability zone

The range of values k_m^2 for which $h(k_m^2)$ is negative is

$$2\beta_D d\, k_{1,2}^2 = [\ \alpha_R\ [d\ f_u + g_v] - [1+d]S\ ] \mp \sqrt{(\alpha_R\ [d\ f_u + g_v] - [1+d]S)^2 - 4d\left[\alpha_R^2|\mathbb{A}| - \alpha_R\ tr(\mathbb{A})\ S + S^2\right]}. \quad (41)$$

In two dimensions, in a rectangular domain $L_x(t) \times L_y(t)$ the condition becomes

$$k_1^2 < k_{nm} = \left(\left(\frac{n}{L_x}\right)^2 + \left(\frac{m}{L_y}\right)^2\right)^2 \pi^2 < k_2^2, \quad (42)$$

with n and m positive integers. It can be verified that for $S = 0$ (absence of growth), the standard conditions are recovered.

References

[1] Murray JD. Spatial models and biomedical applications. Mathematical Biology. 2003;.

[2] Schnakenberg J. Simple chemical reaction systems with limit cycle behaviour. Journal of theoretical biology. 1979;81(3):389–400.
