# Supplementary material for: Development of whole-limb skeletal patterning through the coordination of growth and self-organization models
Source: PLoS Comput Biol. 2026 Jul 7;22(7):e1014348. doi: 10.1371/journal.pcbi.1014348 (PMC13384404; doi:10.1371/journal.pcbi.1014348)
Supplement: Attachment — Submitted filename: PlosGPP_Answer2Reviewers.pdf [file pcbi.1014348.s010.pdf]

“Development of whole-limb skeletal patterning through the coordination of growth and self-organization models”

Answer to reviewers

1 Reviewer # 1

Use of the term “prediction”

I find the use of the term “prediction” in the Results section to be somewhat overstated. The authors employ this term frequently in the subsection titles (e.g., “Predicting whole limb mouse patterning [...]” and “Integrating experimental data to predict [...]”) as well as in several figure captions. In my view, the manuscript presents simulations that are consistent with biological observations and serve primarily an explanatory, rather than predictive, purpose. I therefore recommend replacing the term “predict” with “simulate”, and “predicting” with “simulating”, to more accurately reflect the nature of the work.

We have revised the manuscript to replace most instances of “predict” with “simulate”, particularly in section titles and figure captions, to better reflect the explanatory emphasis of the present work. We note, however, that our framework does have predictive capability in the sense that it enables exploration of how variations in growth and signaling parameters influence pattern formation.

Model presentation

In the first part of the Results, the authors introduce their model. I believe this section would benefit from additional detail to enhance clarity. In particular, further explanation is needed when presenting the two state variables employed in the model (u and v):

“Within this framework, the variable u represents the specification of the skeletal elements (i.e., differentiation to cartilage). The system follows activator–substrate kinetics, with the second variable v acting as a substrate that is spatially out of phase with u.”

This clarification is important because u is consistently visualized throughout the manuscript, while the role of v may remain unclear to readers, who may wonder why two state variables are necessary if only one appears central to the analysis. To address this, I recommend including a visual representation of both u and v—potentially as a supplementary figure—to illustrate their spatial relationship.

We agree that this and the following aspects were not sufficiently clear in the original manuscript. We have included a visualization of both fields (see Fig. 2A) to illustrate their spatial interplay and clarified their relationship in the text.

As can be observed in the contour plot of v, the pattern mimics the one of u, but with opposite distribution of valleys and hills. These relative values of u and v are well-known for the Schnakenberg model employed with two components (the minimum necessary for generating spatial patterns), which prompted us to omit the v-patterns when displaying the results our simulations.

l 159–166

> *Vector* $\mathbf{R}(\mathbf{c})$ *corresponds to the reaction term with an activator-substrate kinetics, specifically Schnakenberg kinetics [39],*
>
> $$\mathbf{R}(u, v) = \begin{bmatrix} k_1 - u + u^2 v \\ k_2 - u^2 v \end{bmatrix}. \tag{7}$$
>
> *The reaction term represents the cellular mechanisms by which specification information is interpreted and acted upon. This may include transcriptional activation of genes, signal transduction cascades, or other intracellular processes that regulate cell fate decisions. The variable* $v(\mathbf{x}, t)$ *acts as a substrate that is spatially out of phase with the activator* $u(\mathbf{x}, t)$ *(Fig. 2A), such that regions of high* u *coincide with regions of low* v*, and vice versa.*

Additionally, providing concrete biological examples of what these fields might represent in a developing limb would strengthen the reader's understanding. While I acknowledge that u and v are general abstractions related to osteogenesis, anchoring them to plausible biological interpretations would improve conceptual accessibility.
We have incorporated explicit biological interpretations of u and v in the revised manuscript, linking them to *Sox9*-positive and *Sox9*-negative cell populations to better ground the model in a developmental context.

l 168–174

> *Within the context of our work, the variable* u *represents the specification of the skeletal elements, i.e., differentiation to cartilage. High values of* u *simulate Sox9-positive cells, whereas high values of* v *correspond to Sox9-negative cells. Consequently, the patterns of* u *generated in a forelimb model correspond to the skeletal elements: humerus, radius, ulna, and digits, which will be the focus of visualization for the remainder of this paper.*

A further point requiring elaboration concerns the reaction term described in the Methods (lines 405–407): "The reaction–diffusion equation in a growing domain, given in (1), involves a two-variable vector $c \in R^2$ with a Schnakenberg reaction term R [75], and is solved using the finite element method.". I recommend that the authors explicitly present the equations defining this reaction term to clarify the interactions between u and v.
We have added the explicit equation (7) defining the Schnakenberg reaction term $\mathbf{R}$ in the Methods, clarifying the interactions between u and v (see gray text above).

It may also be useful to briefly comment on alternative reaction terms that could have been considered, either at this stage or in the Discussion.
We have added a brief comment on this in section "Reaction-diffusion equation on a growing domain", where we point out the generality of the model with respect to other models such as the Gierer-Meinhardt:

l 166–168

> *Schnakenberg kinetics were chosen as a well-established and widely used non-linear instance of activator-substrate dynamics, though other out-of-phase reaction terms could serve as suitable alternatives.*

Finally, I suggest revising the section of the Results that discusses the spatial variations of α_R and β_D:
"As α_R increases distally, the wavelength shortens, allowing a transition from one stripe (humerus) to two stripes (radius and ulna) and eventually to multiple stripes (digits). An increase in α_R

from proximal to distal suggests that the reaction contributes more to patterning, relative to growth, in distal segments. The parameter β_D affects the orientation of the stripe-like patterns by controlling the relative weight of diffusion to growth at the distal tip of the limb bud." This description is difficult to follow without visual support. I encourage the authors to include a figure, perhaps in the supplementary material, to visually guide the reader through the patterning transitions and parameter effects described.

We agree that this part was not sufficiently clear and we hope that the revised version improves readability. We have addressed this in two ways. First, the sentences describing the effects of α_R and β_D on patterning have been relocated to follow the presentation of the sensitivity analysis figures (Fig. 2), so that the visual support is already in place when these claims are made. Second, we have revised the wording of these sentences for clarity. The updated paragraphs in the first section of the Results now read:

l 325–334

> *These sensitivity analyses suggest that α_R influences the wavelength of the pattern, which determines the number of stripes (skeletal elements) formed. In the typical case, this allows a transition from one stripe (humerus) to two stripes (radius and ulna) and eventually to multiple stripes (digits). As the value of α_R increases at the autopod, more digits are formed (Fig.4B). Increasing α_R from 4 to 12 increases the number of digits from 2 to 6. Even though PI is defined along the proximal-distal axis through the spatial modulation of α_R, the resulting pattern also reflects anterior-posterior organization of the number of skeletal elements. This highlights how PD inputs can influence AP patterning outcomes and reinforces the known interdependence between the two axes [46, 47].*

l 345–349

> *Parameter β_D (the relative weight of diffusion to growth) affects the orientation of the radial stripe-like pattern of the digits. With a progressive increase in β_D, the pattern gradually orients radially (Fig.4D), with higher values producing a more ordered digit-like arrangement. This highlights the critical role of β_D in influencing digit patterning, similar to the AER [49, 50].*

Clarification on the role of mice data

In the first section of the Results, the authors apply the previously introduced GRD framework to simulate limb embryogenesis in mice. In Fig. 1B–C, they assess two theoretical models of limb formation (Progressive Positional Identity and Early Positional Identity), demonstrating that the former provides a better description of the developmental process. In Fig. 2, they again draw on mouse data to examine how different spatial profiles of α_R and β_D influence the resulting patterning. However, the importance of the mouse data does not come across as clearly as it could. Greater emphasis on this point would help convey not only the generalizability of the model, but also strengthen the transition to the subsequent section, which begins: "To test the robustness of the GRD framework [...], we applied it to the axolotl salamander."

To improve clarity and flow, I recommend revisiting the organization of this portion of the Results. One possible approach would be to divide the section into two subsections: the first offering a general overview of the model (corresponding roughly to current Lines 87–143), and the second discussing the simulation results presented in Fig. 1B–C and Fig. 2. In the second subsection (or second part, should the authors prefer not to formally divide the text), it would be helpful to more explicitly emphasize that these simulations aim to recapitulate limb development in mice. I also suggest adding biological context that connects the simulated outcomes to known features of murine limb morphogenesis. For example, an opening sentence

might read:

"To investigate how the GRD framework captures the temporal evolution of positional identity during limb morphogenesis, we employed developmental data from the mouse model [...]". Such clarification would guide the reader more effectively and underscore the relevance of these results.

Motivated by these comments and those of reviewer #3, we have substantially reorganized the methods and results section. The first section of the results adds an explicit statement that clarifies that the mouse is used as the reference organism:

l 286–288

> *To explore how the GRD framework captures skeletal patterning during limb morphogenesis, a classical model organism in developmental biology is used: the mouse (Fig.3).*

Additionally, this section comments on a sensitivity analysis of the parameters on the resulting patterns in the limb mouse, and also relates them with the experimental observations.

Clarification on developmental stages

At line 203, the authors refer to developmental stages 44 to 47 without providing an accompanying explanation. It would be helpful to briefly clarify what these stages represent or to include an appropriate reference where readers can find additional information.
We have added a reference to clarify the developmental stages mentioned (Nye et al. (2003) [34]). In addition, we have added a correspondence between stages and days in Fig. 1 (previously Fig. 4) of the resubmitted version to provide a practical reference for readers.

Clarification of Fig. 3D

Figure 3D (left panel) presents the distribution of Hoxa 9, 11, and 13 in the developing limb of the axolotl. However, it is very hard to see the distribution of Hoxa11 and Hoxa13. Moreover, it is not clear what the green shade represent. I suggest authors to clarify the interpretation of this image.

We have clarified the caption of Fig. 3D (now Fig. 5D), to make the color coding and the interpretation of the green shade explicit. The caption now reads

Fig. 5

> ***(D)*** *Composite representation of positional information (from Hoxa genes), distal signaling cues (from Wnt genes), and skeletal patterning at developmental stage 46. Note: Hoxa9, Hoxa11, and Hoxa13 are shown in cyan, blue, and yellow respectively; their co-expression results in mixed colours, such that regions where Hoxa13 overlaps with Hoxa9 and Hoxa11 appear green.*

End of the results

The authors conclude the results section with the following sentence:

"The spatial correspondence between the simulation output (Fig.3F, right) and experimental marker expression (Fig.3E) demonstrates that the underlying developmental logic captured by the GRD model is conserved and extensible beyond mouse limb development, without requiring structural modifications to the model itself."

While I agree on the first part of the sentence, I don't know if I agree on the last part (in red). The overlap between the simulations and the experimental data is clear but not striking, and it seems unrealistic to not envision even a small model modification in the future. Moreover,

I don't think it is necessary to state the relevance of the presented approach. I suggest the authors to remove the last part of the sentence, or to replace it with a different text.

We agree that the final part of the sentence could be interpreted as overstated and have removed it in this revised version. Our intention was to refer to the underlying framework or key hypotheses, which we do not expect to require fundamental modification, although refinements may be needed for specific applications.

Reviewer # 2

In "Development of whole-limb skeletal patterning through the coordination of growth and self-organization models", the authors introduce a generalised method to address the beginning of cartilaginous formations in animal limbs, exemplifying with the development of limbs in axolotls, chicken and mice. The document is clear and well written and the model is well explored. The work is relevant and I support its publication in PLoS Computational Biology provided the authors address the following comments.

1. Using partial differential equations to describe pattern formation in limbs is a classical topic in Mathematical Biology. That is correctly reflected in the references of the manuscript, with about 75% of the 76 references being older than 10 years. In this context, the document should be clear regarding both the novelty of the work and the recent (last 5 years) advances in the field that prompted a novel perspective on this topic. Therefore the authors should expand the paragraph between lines 42 and 58 to better justify these two points.

   We agree that the Introduction required clearer positioning of the work within the recent literature. We have substantially revised it to better articulate the novelty of our approach and situate it within recent advances in the field. In particular, we have expanded the paragraph between lines 70 and 956 to address both points raised by the reviewer.

   Regarding recent advances, we have added references to reflect progress made in the last five years, including work on Turing systems under domain growth (Waters et al. [22]; Krause et al. [23]; Krause et al. [25]), and modeling of limb development (Glimm et al. [32]; Newman et al. [3]), digit patterning (Grall et al. [15]).

2. The authors explore the effect of several parameters and indicate that patterning is particularly sensitive to alterations in the rigidity distribution within the limb. However, the resilience of the results to parameter alteration is not quantified. The authors should quantify the resilience of the observed patterns (e.g. by including a sensitivity analysis of individual parameters).

   We have extended the explanation of our sensitivity analysis by being more quantitative. While a formal quantitative metric of robustness is not introduced, the parameter sweeps in the sensitivity study allow us to assess the stability of pattern topology across the explored ranges. The updated paragraph now reads

   l 325–334

   > *These sensitivity analyses suggest that α_R influences the wavelength of the pattern, which determines the number of stripes (skeletal elements) formed. In the typical case, this allows a transition from one stripe (humerus) to two stripes (radius and ulna) and eventually to multiple stripes (digits). As the value of α_R increases at the autopod, more digits are formed (Fig.4B). Increasing α_R from 4 to 12 increases the number of digits from 2 to 6. Even though PI is defined along the proximal-distal axis through the spatial modulation of α_R, the resulting pattern also reflects anterior-posterior organization of the number of skeletal elements. This highlights how PD inputs can influence AP patterning outcomes and reinforces the known interdependence between the two axes [46, 47].*

l 345–349 *Parameter β_D (the relative weight of diffusion to growth) affects the orientation of the radial stripe-like pattern of the digits. With a progressive increase in β_D, the pattern gradually orients radially (Fig.4D), with higher values producing a more ordered digit-like arrangement. This highlights the critical role of β_D in influencing digit patterning, similar to the AER [49, 50].*

l 350–354 *A sensitivity analysis on the kinetic parameters k_1 and k_2 of the Schnakenberg reaction term and on the diffusivity ratio d is provided in Fig.4E. The stylopod and zeugopod patterns remain relatively unaffected compared to the autopod, which as expected, is more strongly influenced by reaction-diffusion dynamics. The patterns overall show robustness to these parameter variations.*

3. In line 136, the authors refer that β_D affects the orientation of the strip-like pattern. We do not observe that the lines can be oriented diagonally in the limbs. So the authors should clarify how does this parameter affect the pattern orientation.
We agree that this point required clarification. We have revised the text to indicate that the term "orientation" refers to whether the pattern forms elongated stripes, as expected for digit-like elements, or isotropic blob-like patterns with no directional preference, as observed in the absence of β_D. To make this distinction clearer, we have moved the relevant supplementary figure to the main text (current Fig. 4D) and improved the visualization to better illustrate how β_D affects stripe orientation.

l 345–349 *Parameter β_D (the relative weight of diffusion to growth) affects the orientation of the radial stripe-like pattern of the digits. With a progressive increase in β_D, the pattern gradually orients radially (Fig.4D), with higher values producing a more ordered digit-like arrangement. This highlights the critical role of β_D in influencing digit patterning, similar to the AER [49, 50].*

4. In figure 1 the authors should identify the limbs positions in the bottom line of the B plate.
We have clarified the orientation of the limb in the caption of panel A, which now includes the sentence "The mice limb is oriented along the PD/AP axis."

Fig. 3 *(**A**) Vector $\mathbf{c}$ corresponds to the two out-of-phase concentrations, u and v, where high values of u indicate differentiation of skeletal elements through cartilage condensation. The convective term $S\mathbf{c}$ captures the effect of growth, with S representing the growth rate. The diffusion term is $\beta_D \mathbb{D} \nabla_x^2 \mathbf{c}$, and the reaction term is $\alpha_R \mathbf{R}(\mathbf{c})$. α_R and β_D indicate the contribution of reaction and diffusion relative to growth in the system. Growth, reaction and diffusion refer to tissue expansion, information regulation and signaling communication. The mice limb is oriented along the PD/AP axis.*

5. In general, the size of the font in all images is too small and hard to read when the images are inserted in the text. Authors should increase font size in the images. Moreover, the diagrams and data in Image 3 are very small. The image would benefit from a re-organization to occupy at least half a page (instead of a third).
We agree with the reviewer and we have increased the font size in all figures to improve

readability. For Figure 3 specifically (now Figure 5), we have also increased the overall size of the figure so that it now occupies half a page.

6. Better explain (describe premises and results in detail) the simulations carried out in Figure 2C (paragraph 179-183 is not sufficiently clear).
   We have addressed the reviewer's concerns and the updated the paragraph describing the simulations carried out in Figure 2C (now Figure 4C), to more clearly describe the premises and results. The extended paragraph now reads

   l 335–344

   > *To further explore α_R (the relative weight of reaction to growth) in segment identity, we tested whether individual limb segments could be selectively removed or shortened by shifting the transition points between the different α_R values (cyan, blue and yellow regions in Fig.4C). We applied this to both the stylopod and the zeugopod by shifting the transition point from the cyan region to the blue one. In both cases, shortening a segment is achievable, consistent with biological experiments [48], but it simultaneously alters autopod patterning. The framework therefore cannot completely remove or shorten the stylopod or zeugopod without affecting the autopod, reflecting the interconnected nature of limb segments where changes to one region impact the development of others.*

7. Could the authors discuss if the patterns themselves could be used influence the limb rigidity? I.e. the model could be started with constant rigidity, and then the calculated patterns could set the locations for high tissue rigidity. Then the space-dependent growth rates and the patterns could be calculated again in a self-consistent manner. Would that be a reasonable approach for a future work?
   This is indeed a compelling direction, and we fully agree on its potential. We have added a more explicit discussion of this point in the Discussion section, where we now state

   l 491–496

   > *A particularly promising avenue would be to couple the model self-consistently. One possible approach would be to use the resulting patterns to inform local tissue rigidity, which in turn feeds back into the growth and patterning computations. Exploring this feedback loop between pattern formation and tissue growth could provide interesting insights into the mechanisms underlying limb morphogenesis.*

   We look forward to exploring this in future work.

8. The reaction term used should be described (and discussed) in the main text, and not just in the Supporting Information.
   We have moved the description and discussion of the reaction term to the main text, within the Methods section. The reaction term is now explicitly given, along with a brief discussion of the choice of Schnakenberg kinetics and the relevance of the out-of-phase relationship between the two variables. The updated Methods section now reads

l 159–173

Vector $\mathbf{R}(\mathbf{c})$ *corresponds to the reaction term with an activator-substrate kinetics, specifically Schnakenberg kinetics [39],*

$$\mathbf{R}(u, v) = \begin{bmatrix} k_1 - u + u^2 v \\ k_2 - u^2 v \end{bmatrix}. \tag{7}$$

The reaction term represents the cellular mechanisms by which specification information is interpreted and acted upon. This may include transcriptional activation of genes, signal transduction cascades, or other intracellular processes that regulate cell fate decisions. The variable $v(\mathbf{x}, t)$ *acts as a substrate that is spatially out of phase with the activator* $u(\mathbf{x}, t)$ *(Fig. 2A), such that regions of high* u *coincide with regions of low* v*, and vice versa. Schnakenberg kinetics were chosen as a well-established and widely used non-linear instance of activator-substrate dynamics, though other out-of-phase reaction terms could serve as suitable alternatives. Within the context of our work, the variable* u *represents the specification of the skeletal elements, i.e., differentiation to cartilage. High values of* u *simulate Sox9-positive cells, whereas high values of* v *correspond to Sox9-negative cells. Consequently, the patterns of* u *generated in a forelimb model correspond to the skeletal elements: humerus, radius, ulna, and digits, which will be the focus of visualization for the remainder of this paper.*

Reviewer #3

In this work, the authors present a methodology to evaluate the development of a limb. They propose a methodology based on growth mechanics and molecular signaling based on reaction-diffusion equations. They show the performance of their methodology in 2 cases: in mice development and in axolots' development, the last based on experimental images.

Despite being an interesting topic, I have my concerns that I would like to be addressed before recomending its publication.

Major points

1. In my opinion, the organisation of the manuscript does not benefit from the 'methods at the end' approach. The concepts are mentioned at the beginning of the Results section, but it is difficult to follow as the details specified in the Methods section are at the end of the paper. The first subsection of the 'Results' section provides an overview of the model. In my opinion, it doesn't make much sense to provide a brief summary here and then expand on it later in the 'Methods' section. This makes it more difficult to follow the methods, especially since the main equation (eq. 1) is introduced at the beginning of the manuscript. In the discussion section, some limitations are mentioned, but it is difficult to understand these if the relevant concepts have not yet been introduced.
   We agree with this suggestion. The manuscript has been reorganized so that the Methods section now precedes the Results section. New section "Materials and Methods" introduces all the necessary equations and parameters, and develops the non-dimensionalisation that gives rise to the discussed parameters α_R and β_D, jointly with the definitions of reference times T_G, T_R and T_D. We believe that the current ordering makes the article clearer.

2. The results focus on showing the performance of the methodology in both mice and axolotls. The first subsection shows the model and the results for the mice, and the second subsection shows the results for the axolotls. The incorporation of the experimental data in the axolotl results is addressed. However, it is unclear what the initial conditions were for the mice experiments. It looks confusing to include the model alongside the mice results and the experimental data inputs alongside the axolotl results.
   Indeed, this aspect was not sufficiently clear in the original manuscript and we have addressed it in two ways. First, we have added an explicit clarifying statement at the opening of the mouse results section to better frame its scope

   > *l 286–288* *To explore how the GRD framework captures skeletal patterning during limb morphogenesis, a classical model organism in developmental biology is used: the mouse (Fig.3).*

   Second, we have reframed the mouse results section to emphasize its sensitivity analysis nature, making it clearer that the mouse section explores the parameter space of the model, while the axolotl section demonstrates the incorporation of experimental data.

3. I am finding it difficult to understand the parameters α and β. I understand that they define the importance of the reaction and diffusion processes, and that they can be varied in order to evaluate different scenarios. However, they are defined in L442 later in the methods. It is unclear to me whether these parameters are simply the result of non-dimensionalisation or whether they are defined beforehand to test different hypotheses. As I understand it, the authors aim to work with these two parameters, normalising the

equations in order to do so. Is this correct?

We acknowledge that this point was not sufficiently clear in the original manuscript. The reviewer is correct: α_R and β_D arise naturally from the non-dimensionalisation of the governing equations. By moving the Methods section before the Results, as suggested by the reviewer, the non-dimensionalisation and the definition of these parameters are now introduced before they are used, which we hope makes their origin and meaning clearer to the reader.

4. Regarding the results in mice, I am not sure how growth is evaluated. Are there images that support the growth plotted in Fig. 1? Could the authors also plot this data?

   The growth for the mouse case is derived from experimental images in the same way as for the axolotl, as described in the Methods section (Derivation of growth from experimental images). Specifically, the limb outline for the mouse was obtained from Musy et al. (2018), from which the growth rate is computed. To make this clearer, we have added a supplementary figure 1A showing the growth rate derived from the mouse data.

5. I find the section starting in L404 in the methods section difficult to follow. Firstly, since equation 1 was introduced so early on, it would be helpful to have it closer to this section. Too many parameters are introduced and, with careful reading, one can follow them. In any case, I do not understand where the relation a_c comes from.

   This aspect has been improved by placing the Methods section before the Results, so that all elements of the formulation are now introduced in a consistent and continuous manner, which improves readability. Regarding a_c, we have added a clarifying sentence, that reads

   > *l 110–113* *The growth velocity at the current configuration is given by*
   >
   > $$\mathbf{a}(\mathbf{x}, t) = \frac{d\mathbf{x}}{dt},$$
   >
   > *where the function* $\mathbf{a}(\mathbf{x}, t)$ *is considered to be known and twice continuously differentiable.*

   Our expression of a_c, now given in equation (9) of the submitted manuscript, aims to define a characteristic growth velocity from reference lengths and times, mimicking the previous expression,

   > *l 189–192* *The characteristic timescale* T_G*, which is associated with domain expansion due to growth, is defined as*
   >
   > $$T_G = \frac{L_0}{a_c},$$
   >
   > *where the characteristic growth velocity*
   >
   > $$a_c = \frac{L_f - L_0}{t_f}$$
   >
   > *has been defined.*

6. What is the purpose of the non-dimensionalisation? Is it to enable comparison with experimental data?

We have added a clarifying sentence at the opening of the non-dimensionalisation section to explicitly state its purpose:

> l 203–205 *The process of non-dimensionalization captures the interactions and interdependencies within the system by identifying the key dimensionless groups that govern the system.*

Furthermore, as the reviewer correctly suggests, by choosing the growth timescale T_G as the characteristic timescale, the resulting dimensionless parameters can be directly informed by and compared to experimental data.

> l 216–218 *One advantage of this approach is that the explicit knowledge of T_G enables straightforward conversion of nondimensional results back into dimensional units, allowing direct comparison with experimental data.*

> l 244–252 *For example, in axolotl limb development, experimental data indicate a final apparent strain $\varepsilon_f = 3.95$ over a period of 12 days. This implies a characteristic growth timescale $T_G = t_f/\varepsilon_f \approx 3.03$ days. Based on the range of α_R values used in our simulations (from 1 to 30), the corresponding reaction timescales $T_R = T_G/\alpha_R$ span from 3.03 days down to 0.101 days. Similarly, the diffusion timescales $T_D = T_G/\beta_D$, with β_D up to 15, span from 3.03 days down to 0.202 days. These estimates demonstrate how the non-dimensional framework allows re-dimensionalization of the system and facilitates interpretation of quantities of interest across biological contexts.*

Importantly, the dimensionless parameters α_R and β_D indicate respectively the importance of the reaction and diffusion processes with respect to the growth process in the different regions of the limb, that is, the contour plots of these parameters provide a clear readout of their contribution.

7. As can be seen in Fig. 6A, the results are highly dependent on tissue rigidity. Different K maps lead to completely different results. How is this K value determined? Is it based on experimental data? Could the authors provide further information on how to address this dependence?
The parameter K is a numerical parameter used to approximate the growth field. In the absence of direct experimental measurements of tissue growth, we use a linear elastic approximation to derive the growth field from the observed shape changes of the limb, and the growth rigidity K arises as part of this approximation (Paragraph *Derivation of growth from experimental images*). Were direct measurements of the growth field available, this approximation (and K along with it) would not be needed. For now, we assume K to be homogeneous as a simplification, and as shown in the supplementary material Fig.S1 and Fig.S3, varying K leads to different results, which further motivates the need for experimental growth data in future work.

8. Overall, I feel that the model is too sensitive to the choice of parameters, and that too many different results can be achieved by changing the main parameters. Therefore, I can't really see the point.
We understand the reviewer's concern. The sensitivity of the model to α_R and β_D is not a limitation but rather a feature of the framework. The central claim of this work is that these two parameters, by capturing the relative contributions of reaction and diffusion compared to growth, are sufficient to recapitulate the diversity of skeletal patterning

outcomes observed across species and developmental stages. Crucially, these parameters are not chosen freely: α_R is informed by Hoxa gene expression along the proximodistal axis, and β_D reflects AER signaling activity. The fact that biologically motivated choices of these parameters reproduce the known skeletal patterns of both the mouse and the axolotl is precisely the validation we offer. The sensitivity analyses presented in Fig. 2 of the resubmitted version are intended to map the parameter space systematically, not to suggest that any combination of parameters is equally valid.

Some other comments are:

- Maybe this field is not familiar to me, but concepts such as positional information are not understood. I understand that L23–41 talks about PI and that Turing systems are discussed later. I would appreciate a little more explanation on these topics.
  We have reorganized the introduction and added an explicit definition of positional information (PI) at the beginning of the relevant paragraph in the Introduction, so that the concept is clearly introduced before it is used. The paragraph now reads

  l 30–40

  > *Two complementary theoretical frameworks have guided computational models of limb patterning. In the positional information (PI) framework, diffusible morphogens establish concentration gradients that assign spatial identity to cells, which then differentiate accordingly [6]. Key signaling centers — the Apical Ectodermal Ridge (AER), providing distal cues guiding PD outgrowth [7, 8], and the Zone of Polarizing Activity (ZPA), controlling AP identity [6, 1] — act as morphogen sources. Hox gene expression provides a molecular readout of positional identity along the PD axis [9]. While positional information explains how cells acquire spatial identity, it does not readily account for how discrete, periodic skeletal elements emerge from a continuous tissue. Reaction-diffusion (Turing) systems address this gap by describing how interactions between two or more morphogens through coupled partial differential equations can spontaneously generate stable spatial patterns [10].*

- L69-70: Those ‘significant contributions’ could be summarised briefly.
  The discussion of these papers has been moved to the Discussion and the summary of these findings now reads

  ll 472–479

  > *Despite this simplified starting assumption, the GRD model generates anisotropic and inhomogeneous growth rate patterns as a result of the boundary conditions (Fig.1B,C). However, studies have shown that growth in the developing limb has inherent directional biases and regional variations [38, 66, 67, 68]. Our preliminary tests with different growth rate distributions show that the specification pattern is significantly impacted by these, both on the rectangular domain (Fig.S2, Fig.S3) and on the developing mouse limb (Fig.S1).*

- Fig. 1 caption: What does ‘out-of-phase’ mean?
  We have added a clarifying sentence in the Methods section to define the term

> l 164–173 *The variable $v(\mathbf{x}, t)$ acts as a substrate that is spatially out of phase with the activator $u(\mathbf{x}, t)$ (Fig. 2A), such that regions of high u coincide with regions of low v, and vice versa. Schnakenberg kinetics were chosen as a well-established and widely used non-linear instance of activator-substrate dynamics, though other out-of-phase reaction terms could serve as suitable alternatives. Within the context of our work, the variable u represents the specification of the skeletal elements, i.e., differentiation to cartilage. High values of u simulate Sox9-positive cells, whereas high values of v correspond to Sox9-negative cells. Consequently, the patterns of u generated in a forelimb model correspond to the skeletal elements: humerus, radius, ulna, and digits, which will be the focus of visualization for the remainder of this paper.*

Additionally, we have added a panel in Fig. 2A (previously Fig. 5B) to visually illustrate this relationship.

- Fig. 1A: Could the authors state the same equation as equation 1 and explain why they include the dependency on $\mathbf{c}$ in the term $\mathbf{R}$ but not the other dependencies?
We have removed all dependencies in the equation of Fig. 1 (now Fig. 3). Also, in equation (1), now equation (6), we have removed the dependency of $\mathbf{R}$ on the coordinates $\mathbf{x}$ and time t, which was unnecessary.

- L127–129: I don't understand how α_R and β_D change over space and time. I later understood that this is based on the data from Fig. 3, but I still don't understand how these parameters are set.
The values of α_R and β_D are set by adapting and smoothing the contours of the experimental images. We have added two clarifying sentences that read as follows

> l 306 – 311 *To simulate this scenario, α_R evolves from a single uniform value into two and then three distinct regions using a sigmoid function normalized to the final domain length . The different regions correspond to the limb segments and reflecting the gradual establishment of positional identity. Similarly, β_D is modulated by linearly increasing its distribution over time at the distal tip.*

- L214: Could the authors explain why their results match those of others?
We have revised the sentence to make the comparison more explicit

> l 381 – 386 *We observed that Hoxa9 was expressed broadly throughout the mesenchyme from stage 44 onward. Hoxa11 was expressed from the middle portion of the limb bud to the distal tip from stage 45 to stage 47. Hoxa13 first appeared at the distal tip around stage 45.5 and remained restricted to the autopod region through subsequent stages. These results are consistent with previously reported spatiotemporal expression patterns of Hoxa genes during axolotl limb development [60].*

- In figures 1 and 2, using different colour maps for PI and AER would improve readability.
We have intended to use different color maps for each sequence. Although we recognize that for some plots these are not fully distinct, modifying the map would require modifying all the subsequent plots, for a relative minor gain. Nonetheless, we do retain the suggestion for further works.

- Fig. 3: Are these fluorescence images 2D?
  We have made this clearer in the main text, which now reads

  l 375 – 377

  > *Whole-mount samples were imaged in 3D, reoriented, and a representative 2D section capturing spatial gene expression patterns along the PD and AP axes was extracted for analysis (Fig.5C).*

- Could the fluorescence of the bottom layers also be reflected in the image? What is the process of converting experimental data (A, B and E) to computational input data (F)?
  We have added a clarifying sentence to make this process explicit. The updated paragraph now reads

  l 404 – 415

  > *Using the experimentally informed geometries and spatial inputs, we ran numerical simulations of the GRD framework to predict sites of skeletal patterning across developmental stages (Fig.5F). In these simulations, α_R and β_D were assigned to reflect the distributions of Hox gene expression and distal signaling factors, respectively. Similar to the images in Fig.5A, the segments' identities are acquired progressively: the stylopod region (cyan) is established from the start, followed by the emergence of the zeugopod domain (blue) around stage 45.5, and finally the autopod (orange) around the end of stage 46 to stage 47. Distal cues are applied at the distal tip from as early as stage 44–45 and persist throughout the limb development. The conversion from experimental data (Fig.5A, B) to computational inputs (Fig.5F) therefore relies on qualitatively matching the spatiotemporal profiles of α_R and β_D (the relative weight of reaction and diffusion to growth) to the observed gene expression domains.*

- What is the definition of 'stage'? Is it one day?
  Developmental stages are defined by morphological criteria rather than by time. As such, a stage duration can vary between individuals and experimental conditions. We have added a correspondence between stages and days in Fig. 1 (previously Fig. 4) to provide a practical reference for readers, but this mapping is approximate. We have clarified this point in Fig. 1 legend with a reference that reads

  Fig. 1

  > *Stereo microscopy images were taken daily for a week from the same animal. An approximate correspondence between days and the axolotl limb developmental stages is provided for a practical temporal reference [34].*

- In subfigure D, could the authors plot Hoxa13 in yellow? What about Fgf8?
  We have added a Note in the caption on the visualization of Hoxa13

  Fig. 5

  > *(**D**) Composite representation of positional information (from Hoxa genes), distal signaling cues (from Wnt genes), and skeletal patterning at developmental stage 46. Note: Hoxa9, Hoxa11, and Hoxa13 are shown in cyan, blue, and yellow respectively; their co-expression results in mixed colours, such that regions where Hoxa13 overlaps with Hoxa9 and Hoxa11 appear green.*

- L244 and the caption of Fig. 3: sox9 should be in italics.
  We thank the reviewer for catching this. *Sox9* has been italicised throughout the manuscript, including at L244 and in the caption of the corresponding figure.

- L280: typo, respectively.
  We thank the reviewer for their careful reading. The typo has been corrected.

- L287: Fig. 5 is mentioned before Fig. 4.
  The figure is referenced in the Discussion section, where figures are cited in the context of the argument being made rather than in sequential order. We are happy to revise the ordering if preferred.

- L332: Could the authors please specify how the image data is included in the model?

  We use the data in the experimental images in two ways: for approximating plausible distributions of parameters α_R and β_D, and also to define the coordinates of the boundary of the domains, which is used for updating the domain at each time-step, and as described in the caption of current Fig. 1 (previously Fig. 4),

  Fig. 1
  > *The limb outline was manually extracted from these images and meshed using triangular linear elements.*

- L370: is the last X a capital letter?
  Yes, the last X is intentionally capitalized, as it denotes the reference coordinate (the configuration at $t = 0$), whereas x refers to the current spatial position (the configuration at $t > 0$).

- Fig. 4: The mesh is not easily distinguishable. If the authors want to plot this, they should consider an alternative approach. How is the conversion between stage and days?
  We have added to the Fig. 1 a correspondence between the axolotl developmental stages and days.

- L433: What about non-dimensionalisation?
  We thank the reviewer for this suggestion; the paragraph name was changed accordingly.

- L458: maybe add a B to 'Fig. 5'?
  We thank the reviewer for their careful reading. The reference has been updated to Fig. 5B.

- Fig. 6 caption: I don't understand what the mesh has to do here.
  We have revised the caption to make the role of the mesh clearer

  Fig.S3
  > *To assess whether pattern transport is a numerical artifact of the evolving mesh, simulations were repeated without the convective term, using homogeneous (B1) and linear (B2) growth rigidity profiles. The fact that B1 and B2 yield identical results confirms that the patterns are independent of mesh deformation.*

- L508: What is the time increment? Is it 0.0017 days?
  We thank the reviewer for pointing this out. Our units of time are non-dimensional, and we see that the confusion is due to the incorrect use of Δt instead of $\Delta \tau$. We have corrected and completed the sentence.

l 257 – 261 *According to our non-dimensionalization, relative time is given by* $\tau = \varepsilon_f t/t_f$*, and therefore at final time* $\tau_f = \varepsilon_f$*. In our simulations, the number of time increments is given by* $\varepsilon_f/\Delta\tau$*, with* $\Delta\tau$ *the non-dimensional time step. In the mice case, we used* $\Delta\tau = 0.0017$ *and a total number of increments of* 3804 *to achieve the corresponding* $\varepsilon_f \sim 6.5$ *fold lengthening.*
