# Supplementary material for: Development of whole-limb skeletal patterning through the coordination of growth and self-organization models
Source: PLoS Comput Biol. 2026 Jul 7;22(7):e1014348. doi: 10.1371/journal.pcbi.1014348 (PMC13384404; doi:10.1371/journal.pcbi.1014348)
Supplement: Attachment — Submitted filename: Comment_PCOMPBIOL-D-25-01875.docx [file pcbi.1014348.s009.docx]

# Comment on “Development of whole-limb skeletal patterning through the coordination of growth and self-organization models”

## In this paper, Tahar and colleagues present a mathematical framework based on partial differential equations to investigate limb development in tetrapods, with a specific focus on mice and the axolotl. I particularly appreciate the authors’ effective integration of realistic geometries, experimental observations, and classical elastic theory to elucidate the role of cellular proliferation in limb morphogenesis. The manuscript is clearly written, exhibits a coherent narrative flow, and successfully bridges the proposed mathematical model with key biological processes in embryogenesis.

## Although the manuscript is generally well-developed, I believe that a few minor revisions—particularly within the Results section—could further enhance the clarity of the presented concepts. Below, I outline several suggestions aimed at improving the overall quality of the work.

## Code accessibility

The authors state that the code they used is available at <https://gitlab.com/turing-embryogenesis-group/gpp-code.git>, but the link does not provide access to the source scripts. I recommend to ensure the accessibility of the code before publication.

## Use of the term “prediction”

I find the use of the term “prediction” in the Results section to be somewhat overstated. The authors employ this term frequently in the subsection titles (e.g., “Predicting whole limb mouse patterning [...]” and “Integrating experimental data to predict [...]”) as well as in several figure captions. In my view, the manuscript presents simulations that are consistent with biological observations and serve primarily an explanatory, rather than predictive, purpose. I therefore recommend replacing the term “predict” with “simulate”, and “predicting” with “simulating”, to more accurately reflect the nature of the work.

## Model presentation

In the first part of the Results, the authors introduce their model. I believe this section would benefit from additional detail to enhance clarity. In particular, further explanation is needed when presenting the two state variables employed in the model (u and v):

“Within this framework, the variable u represents the specification of the skeletal elements (i.e., differentiation to cartilage). The system follows activator–substrate kinetics, with the second variable v acting as a substrate that is spatially out of phase with u.”

This clarification is important because u is consistently visualized throughout the manuscript, while the role of v may remain unclear to readers, who may wonder why two state variables are necessary if only one appears central to the analysis. To address this, I recommend including a visual representation of both u and v—potentially as a supplementary figure—to illustrate their spatial relationship. Additionally, providing concrete biological examples of what these fields might represent in a developing limb would strengthen the reader’s understanding. While I acknowledge that u and v are general abstractions related to osteogenesis, anchoring them to plausible biological interpretations would improve conceptual accessibility.

A further point requiring elaboration concerns the reaction term described in the Methods (lines 405–407):

“The reaction–diffusion equation in a growing domain, given in (1), involves a two-variable vector c ∈ ℝ² with a Schnakenberg reaction term R [75], and is solved using the finite element method.”

I recommend that the authors explicitly present the equations defining this reaction term to clarify the interactions between u and v. It may also be useful to briefly comment on alternative reaction terms that could have been considered, either at this stage or in the Discussion.

Finally, I suggest revising the section of the Results that discusses the spatial variations of α_R_ and β_D_:

“As α_R_ increases distally, the wavelength shortens, allowing a transition from one stripe (humerus) to two stripes (radius and ulna) and eventually to multiple stripes (digits). An increase in α_R_ from proximal to distal suggests that the reaction contributes more to patterning, relative to growth, in distal segments. The parameter β_D_ affects the orientation of the stripe-like patterns by controlling the relative weight of diffusion to growth at the distal tip of the limb bud.”

This description is difficult to follow without visual support. I encourage the authors to include a figure, perhaps in the supplementary material, to visually guide the reader through the patterning transitions and parameter effects described.

## Clarification on the role of mice data

In the first section of the Results, the authors apply the previously introduced GRD framework to simulate limb embryogenesis in mice. In Fig. 1B–C, they assess two theoretical models of limb formation (Progressive Positional Identity and Early Positional Identity), demonstrating that the former provides a better description of the developmental process. In Fig. 2, they again draw on mouse data to examine how different spatial profiles of α_R_ and β_D_ influence the resulting patterning. However, the importance of the mouse data does not come across as clearly as it could. Greater emphasis on this point would help convey not only the generalizability of the model, but also strengthen the transition to the subsequent section, which begins: “To test the robustness of the GRD framework [...], we applied it to the axolotl salamander.”

To improve clarity and flow, I recommend revisiting the organization of this portion of the Results. One possible approach would be to divide the section into two subsections: the first offering a general overview of the model (corresponding roughly to current Lines 87–143), and the second discussing the simulation results presented in Fig. 1B–C and Fig. 2.

In the second subsection (or second part, should the authors prefer not to formally divide the text), it would be helpful to more explicitly emphasize that these simulations aim to recapitulate limb development in mice. I also suggest adding biological context that connects the simulated outcomes to known features of murine limb morphogenesis. For example, an opening sentence might read:

“To investigate how the GRD framework captures the temporal evolution of positional identity during limb morphogenesis, we employed developmental data from the mouse model [...]”.

Such clarification would guide the reader more effectively and underscore the relevance of these results.

## Clarification on developmental stages

## At line 203, the authors refer to developmental stages 44 to 47 without providing an accompanying explanation. It would be helpful to briefly clarify what these stages represent or to include an appropriate reference where readers can find additional information.

## Clarification of Fig. 3D

Figure 3D (left panel) presents the distribution of Hoxa 9, 11, and 13 in the developing limb of the axolotl. However, it is very hard to see the distribution of Hoxa11 and Hoxa13. Moreover, it is not clear what the green shade represent. I suggest authors to clarify the interpretation of this image.

## End of the results

The authors conclude the results section with the following sentence:

“The spatial correspondence between the simulation output (Fig.3F, right) and experimental marker expression (Fig.3E) demonstrates that the underlying developmental logic captured by the GRD model is conserved and extensible beyond mouse limb development, without requiring structural modifications to the model itself.”

While I agree on the first part of the sentence, I don’t know if I agree on the last part (in red). The overlap between the simulations and the experimental data is clear but not striking, and it seems unrealistic to not envision even a small model modification in the future. Moreover, I don’t think it is necessary to state the relevance of the presented approach. I suggest the authors to remove the last part of the sentence, or to replace it with a different text.
