# Supplementary material for: Development of whole-limb skeletal patterning through the coordination of growth and self-organization models
Source: PLoS Comput Biol. 2026 Jul 7;22(7):e1014348. doi: 10.1371/journal.pcbi.1014348 (PMC13384404; doi:10.1371/journal.pcbi.1014348)
Supplement: Peer review history

# Peer review: Decision Letter 0

**DOI:** 10.1371/journal.pcbi.1014348.r001

**Authors:** Mark Alber, Guillermo Lorenzo

3 Feb 2026

-->PCOMPBIOL-D-25-01875

Development of whole-limb skeletal patterning through the coordination of growth and self-organization models

PLOS Computational Biology

Dear Dr. Ben Tahar,

Thank you for submitting your manuscript to PLOS Computational Biology. After careful consideration, we feel that it has merit but does not fully meet PLOS Computational Biology's publication criteria as it currently stands. Therefore, we invite you to submit a revised version of the manuscript that addresses the points raised during the review process.

Please submit your revised manuscript by Apr 05 2026 11:59PM. If you will need more time than this to complete your revisions, please reply to this message or contact the journal office at ploscompbiol@plos.org. When you're ready to submit your revision, log on to https://www.editorialmanager.com/pcompbiol/ and select the 'Submissions Needing Revision' folder to locate your manuscript file.

Please include the following items when submitting your revised manuscript:

* A letter that responds to each point raised by the editor and reviewer(s). You should upload this letter as a separate file labeled 'Response to Reviewers'. This file does not need to include responses to formatting updates and technical items listed in the 'Journal Requirements' section below.

* A marked-up copy of your manuscript that highlights changes made to the original version. You should upload this as a separate file labeled 'Revised Manuscript with Track Changes'.

* An unmarked version of your revised paper without tracked changes. You should upload this as a separate file labeled 'Manuscript'.

If you would like to make changes to your financial disclosure, competing interests statement, or data availability statement, please make these updates within the submission form at the time of resubmission. Guidelines for resubmitting your figure files are available below the reviewer comments at the end of this letter

We look forward to receiving your revised manuscript.

Kind regards,

Guillermo Lorenzo

Academic Editor

PLOS Computational Biology

Mark Alber

Section Editor

PLOS Computational Biology

**Additional Editor Comments:**

Dear Authors,

Thank you for submitting your manuscript to PLOS Computational Biology. The reviewers have now completed their evaluations. Overall, they consider the study to be potentially suitable for publication in the journal, provided that the concerns raised in their reports are adequately addressed in a revised version.

The reviewers have identified several issues that require attention. In particular, the revision should focus on improving the organization of the manuscript (for example, placing the Methods section before the Results and strengthening the analysis and presentation of the results), providing a sufficiently detailed and clear description of the model and its components, and including a more rigorous analysis of the results (e.g., sensitivity analyses and data that support the model conclusions).

We would therefore welcome the submission of a revised manuscript that carefully addresses these points and the reviewers’ comments in full.

Sincerely,

GL

**Journal Requirements:**

If the reviewer comments include a recommendation to cite specific previously published works, please review and evaluate these publications to determine whether they are relevant and should be cited. There is no requirement to cite these works unless the editor has indicated otherwise.

1) Please ensure that the CRediT author contributions listed for every co-author are completed accurately and in full.

At this stage, the following Authors/Authors require contributions: Soha Ben Tahar, Dareen Bakr, Ester Comellas, Timothy Duerr, James Monaghan, Jose Muñoz, and Sandra Shefelbine. Please ensure that the full contributions of each author are acknowledged in the "Add/Edit/Remove Authors" section of our submission form.

The list of CRediT author contributions may be found here: https://journals.plos.org/ploscompbiol/s/authorship#loc-author-contributions

2) Please upload all main figures as separate Figure files in .tif or .eps format. For more information about how to convert and format your figure files please see our guidelines:

https://journals.plos.org/ploscompbiol/s/figures

3) Some material included in your submission may be copyrighted. According to PLOSu2019s copyright policy, authors who use figures or other material (e.g., graphics, clipart, maps) from another author or copyright holder must demonstrate or obtain permission to publish this material under the Creative Commons Attribution 4.0 International (CC BY 4.0) License used by PLOS journals. Please closely review the details of PLOSu2019s copyright requirements here: PLOS Licenses and Copyright. If you need to request permissions from a copyright holder, you may use PLOS's Copyright Content Permission form.

Please respond directly to this email and provide any known details concerning your material's license terms and permissions required for reuse, even if you have not yet obtained copyright permissions or are unsure of your material's copyright compatibility. Once you have responded and addressed all other outstanding technical requirements, you may resubmit your manuscript within Editorial Manager.

Potential Copyright Issues:

i) Please confirm (a) that you are the photographer of 4A, or (b) provide written permission from the photographer to publish the photo(s) under our CC BY 4.0 license.

4) In the online submission form, you indicated that your data will be submitted to a repository upon acceptance. We strongly recommend all authors deposit their data before acceptance, as the process can be lengthy and hold up publication timelines. Please note that, though access restrictions are acceptable now, your entire minimal dataset will need to be made freely accessible if your manuscript is accepted for publication. This policy applies to all data except where public deposition would breach compliance with the protocol approved by your research ethics board. If you are unable to adhere to our open data policy, please kindly revise your statement to explain your reasoning and we will seek the editor's input on an exemption.

5) Please amend your detailed Financial Disclosure statement. This is published with the article. It must therefore be completed in full sentences and contain the exact wording you wish to be published.

1) State what role the funders took in the study. If the funders had no role in your study, please state: "The funders had no role in study design, data collection and analysis, decision to publish, or preparation of the manuscript."

2) If any authors received a salary from any of your funders, please state which authors and which funders..

If you did not receive any funding for this study, please simply state: u201cThe authors received no specific funding for this work.u201d

6) Please ensure that the files are uploaded in the online submission form in a correct numerical order.

7) Kindly revise your competing statement in the online submission form to align with the journal's style guidelines: 'The authors declare that there are no competing interests.'

**Reviewers' comments:**

Reviewer's Responses to Questions

**Comments to the Authors:**

**Please note here if the review is uploaded as an attachment.**

Reviewer #1: I provided my comment in the attached file.

Reviewer #2: In "Development of whole-limb skeletal patterning through the coordination of growth and self-organization models", the authors introduce a generalised method to address the beginning of cartilaginous formations in animal limbs, exemplifying with the development of limbs in axolotls, chicken and mice. The document is clear and well written and the model is well explored. The work is relevant and I support its publication in PLoS Computational Biology provided the authors address the following comments.

1: Using partial differential equations to describe pattern formation in limbs is a classical topic in Mathematical Biology. That is correctly reflected in the references of the manuscript, with about 75% of the 76 references being older than 10 years. In this context, the document should be clear regarding both the novelty of the work and the recent (last 5 years) advances in the field that prompted a novel perspective on this topic. Therefore the authors should expand the paragraph between lines 42 and 58 to better justify these two points.

2: The authors explore the effect of several parameters and indicate that patterning is particularly sensitive to alterations in the rigidity distribution within the limb. However, the resilience of the results to parameter alteration is not quantified. The authors should quantify the resilience of the observed patterns (e.g. by including a sensitivity analysis of individual parameters).

3: In line 136, the authors refer that \beta_D affects the orientation of the strip-like pattern. We do not observe that the lines can be oriented diagonally in the limbs. So the authors should clarify how how does this parameter affect the pattern orientation.

4: In figure 1 the authors should identify the limbs positions in the bottom line of the B plate.

5: In general, the size of the font in all images is too small and hard to read when the images are inserted in the text. Authors should increase font size in the images. Moreover, the diagrams and data in Image 3 are very small. The image would benefit from a re-organization to occupy at least half a page (instead of a third).

6: Better explain (describe premises and results in detail) the simulations carried out in Figure 2C (paragraph 179-183 is not sufficiently clear).

7: Could the authors discuss if the patterns themselves could be used influence the limb rigidity? I.e. the model could be started with constant rigidity, and then the calculated patterns could set the locations for high tissue rigidity. Then the space-dependent growth rates and the patterns could be calculated again in a self-consistent manner. Would that be a reasonable approach for a future work?

8: The reaction term used should be described (and discussed) in the main text, and not just in the Supporting Information.

Reviewer #3: In this work, the authors present a methodology to evaluate the development of a limb. They propose a methodology based on growth mechanics and molecular signaling based on reaction-diffusion equations. They show the performance of their methodology in 2 cases: in mice development and in axolots’ development, the last based on experimental images.

Despite being an interesting topic, I have my concerns that I would like to be addressed before recomending its publication.

Major points:

In my opinion, the organisation of the manuscript does not benefit from the 'methods at the end' approach. The concepts are mentioned at the beginning of the Results section, but it is difficult to follow as the details specified in the Methods section are at the end of the paper. The first subsection of the 'Results' section provides an overview of the model. In my opinion, it doesn't make much sense to provide a brief summary here and then expand on it later in the 'Methods' section. This makes it more difficult to follow the methods, especially since the main equation (eq. 1) is introduced at the beginning of the manuscript. In the discussion section, some limitations are mentioned, but it is difficult to understand these if the relevant concepts have not yet been introduced.

The results focus on showing the performance of the methodology in both mice and axolotls. The first subsection shows the model and the results for the mice, and the second subsection shows the results for the axolotls. The incorporation of the experimental data in the axolotl results is addressed. However, it is unclear what the initial conditions were for the mice experiments. It looks confusing to include the model alongside the mice results and the experimental data inputs alongside the axolotl results.

I am finding it difficult to understand the parameters α and β. I understand that they define the importance of the reaction and diffusion processes, and that they can be varied in order to evaluate different scenarios. However, they are defined in L442 later in the methods. It is unclear to me whether these parameters are simply the result of non-dimensionalisation or whether they are defined beforehand to test different hypotheses. As I understand it, the authors aim to work with these two parameters, normalising the equations in order to do so. Is this correct?

Regarding the results in mice, I am not sure how growth is evaluated. Are there images that support the growth plotted in Fig. 1? Could the authors also plot this data?

I find the section starting in L404 in the methods section difficult to follow. Firstly, since equation 1 was introduced so early on, it would be helpful to have it closer to this section. Too many parameters are introduced and, with careful reading, one can follow them. In any case, I do not understand where the relation a_c comes from.

What is the purpose of the non-dimensionalisation? Is it to enable comparison with experimental data?

As can be seen in Fig. 6A, the results are highly dependent on tissue rigidity. Different K maps lead to completely different results. How is this K value determined? Is it based on experimental data? Could the authors provide further information on how to address this dependency?

Overall, I feel that the model is too sensitive to the choice of parameters, and that too many different results can be achieved by changing the main parameters. Therefore, I can’t really see the point.

Some other comments are:

- Maybe this field is not familiar to me, but concepts such as positional information are not understood. I understand that L23–41 talks about PI and that Turing systems are discussed later. I would appreciate a little more explanation on these topics.

- L69-70: Those 'significant contributions' could be summarised briefly.

- Fig. 1 caption: What does 'out-of-phase' mean?

Fig. 1A: Could the authors state the same equation as equation 1 and explain why they include the dependency on c in the term R but not the other dependencies?

- L127–129: I don't understand how α_R and β_D change over space and time. I later understood that this is based on the data from Fig. 3, but I still don't understand how these parameters are set.

- L214: Could the authors explain why their results match those of others?

In figures 1 and 2, using different colour maps for PI and AER would improve readability.

- Fig. 3: Are these fluorescence images 2D? Could the fluorescence of the bottom layers also be reflected in the image? What is the process of converting experimental data (A, B and E) to computational input data (F)? What is the definition of 'stage'? Is it one day? In subfigure D, could the authors plot Hoxa13 in yellow? What about Fgf8?

- L244 and the caption of Fig. 3: sox9 should be in italics.

- L280: typo, respectively.

- L287: Fig. 5 is mentioned before Fig. 4.

- L332: Could the authors please specify how the image data is included in the model?

- L370: is the last X a capital letter?

- Fig. 4: The mesh is not easily distinguishable. If the authors want to plot this, they should consider an alternative approach. How is the conversion between stage and days?

- L433: What about non-dimensionalisation?

- L458: maybe add a B to 'Fig. 5'?

- Fig. 6 caption: I don't understand what the mesh has to do here.

- L508: What is the time increment? Is it 0.0017 days?

**********

**Have the authors made all data and (if applicable) computational code underlying the findings in their manuscript fully available?**

The PLOS Data policy requires authors to make all data and code underlying the findings described in their manuscript fully available without restriction, with rare exception (please refer to the Data Availability Statement in the manuscript PDF file). The data and code should be provided as part of the manuscript or its supporting information, or deposited to a public repository. For example, in addition to summary statistics, the data points behind means, medians and variance measures should be available. If there are restrictions on publicly sharing data or code —e.g. participant privacy or use of data from a third party—those must be specified.

Reviewer #1: Yes

Reviewer #2: **No:** Code not available, but sufficiently well described so that it could be implemented by a reader.

Reviewer #3: **No:** link is not provided in the manuscript. Link in the editorial section is not working.

**********

PLOS authors have the option to publish the peer review history of their article (what does this mean?). If published, this will include your full peer review and any attached files.

If you choose “no”, your identity will remain anonymous but your review may still be made public.

**Do you want your identity to be public for this peer review?**  For information about this choice, including consent withdrawal, please see our Privacy Policy.

Reviewer #1: **Yes:** Franco Pradelli, Ph.D.

Reviewer #2: No

Reviewer #3: No

[NOTE: If reviewer comments were submitted as an attachment file, they will be attached to this email and accessible via the submission site. Please log into your account, locate the manuscript record, and check for the action link "View Attachments". If this link does not appear, there are no attachment files.]

**Figure resubmission:**

While revising your submission, we strongly recommend that you use PLOS’s NAAS tool (https://ngplosjournals.pagemajik.ai/artanalysis) to test your figure files. NAAS can convert your figure files to the TIFF file type and meet basic requirements (such as print size, resolution), or provide you with a report on issues that do not meet our requirements and that NAAS cannot fix.-->

After uploading your figures to PLOS’s NAAS tool - https://ngplosjournals.pagemajik.ai/artanalysis, NAAS will process the files provided and display the results in the "Uploaded Files" section of the page as the processing is complete. If the uploaded figures meet our requirements (or NAAS is able to fix the files to meet our requirements), the figure will be marked as "fixed" above. If NAAS is unable to fix the files, a red "failed" label will appear above. When NAAS has confirmed that the figure files meet our requirements, please download the file via the download option, and include these NAAS processed figure files when submitting your revised manuscript.

**Reproducibility:**

To enhance the reproducibility of your results, we recommend that authors of applicable studies deposit laboratory protocols in protocols.io, where a protocol can be assigned its own identifier (DOI) such that it can be cited independently in the future. Additionally, PLOS ONE offers an option to publish peer-reviewed clinical study protocols. Read more information on sharing protocols at https://plos.org/protocols?utm_medium=editorial-email&utm_source=authorletters&utm_campaign=protocols

Attachment Submitted filename: Comment_PCOMPBIOL-D-25-01875.docx

# Author comment: Author response to Decision Letter 1

**DOI:** 10.1371/journal.pcbi.1014348.r002

25 Apr 2026

Attachment Submitted filename: PlosGPP_Answer2Reviewers.pdf

# Peer review: Decision Letter 1

**DOI:** 10.1371/journal.pcbi.1014348.r003

**Authors:** Mark Alber, Guillermo Lorenzo, Mark Alber, Guillermo Lorenzo

21 May 2026

Dear Mx Ben Tahar,

We are pleased to inform you that your manuscript 'Development of whole-limb skeletal patterning through the coordination of growth and self-organization models' has been provisionally accepted for publication in PLOS Computational Biology.

Before your manuscript can be formally accepted you will need to complete some formatting changes, which you will receive in a follow up email. A member of our team will be in touch with a set of requests.

Please note that your manuscript will not be scheduled for publication until you have made the required changes, so a swift response is appreciated.

IMPORTANT: The editorial review process is now complete. PLOS will only permit corrections to spelling, formatting or significant scientific errors from this point onwards. Requests for major changes, or any which affect the scientific understanding of your work, will cause delays to the publication date of your manuscript.

Should you, your institution's press office or the journal office choose to press release your paper, you will automatically be opted out of early publication. We ask that you notify us now if you or your institution is planning to press release the article. All press must be co-ordinated with PLOS.

Thank you again for supporting Open Access publishing; we are looking forward to publishing your work in PLOS Computational Biology.

Best regards,

Guillermo Lorenzo

Academic Editor

PLOS Computational Biology

Mark Alber

Section Editor

PLOS Computational Biology

***********************************************************

Reviewer's Responses to Questions

**Comments to the Authors:**

**Please note here if the review is uploaded as an attachment.**

Reviewer #1: I am happy with the modifications introduced by the authors to address my comments. I think the manuscript reads much better now. A small note that do not require minor revision: Fig.2 appears in low resolution. Before definitive submission, I suggest the authors to increase the resolution of the figure to make it more readable (all the others look good to me).

Reviewer #2: The authors have addressed all my comments. The document has improved significantly. I support the publication.

Reviewer #3: The authors have addressed my comments, therefore I recommend the manuscript for its publication.

**********

**Have the authors made all data and (if applicable) computational code underlying the findings in their manuscript fully available?**

The PLOS Data policy requires authors to make all data and code underlying the findings described in their manuscript fully available without restriction, with rare exception (please refer to the Data Availability Statement in the manuscript PDF file). The data and code should be provided as part of the manuscript or its supporting information, or deposited to a public repository. For example, in addition to summary statistics, the data points behind means, medians and variance measures should be available. If there are restrictions on publicly sharing data or code —e.g. participant privacy or use of data from a third party—those must be specified.

Reviewer #1: Yes

Reviewer #2: **No:** Simulations done with in-house Matlab code not made available.

Reviewer #3: Yes

**********

PLOS authors have the option to publish the peer review history of their article (what does this mean?). If published, this will include your full peer review and any attached files.

If you choose “no”, your identity will remain anonymous but your review may still be made public.

**Do you want your identity to be public for this peer review?**  For information about this choice, including consent withdrawal, please see our Privacy Policy.

Reviewer #1: No

Reviewer #2: No

Reviewer #3: No

# Editor report: Acceptance letter

**DOI:** 10.1371/journal.pcbi.1014348.r004

**Authors:** Mark Alber, Guillermo Lorenzo, Mark Alber, Guillermo Lorenzo

PCOMPBIOL-D-25-01875R1

Development of whole-limb skeletal patterning through the coordination of growth and self-organization models

Dear Dr Ben Tahar,

I am pleased to inform you that your manuscript has been formally accepted for publication in PLOS Computational Biology. Your manuscript is now with our production department and you will be notified of the publication date in due course.

The corresponding author will soon be receiving a typeset proof for review, to ensure errors have not been introduced during production. Please review the PDF proof of your manuscript carefully, as this is the last chance to correct any errors. Please note that major changes, or those which affect the scientific understanding of the work, will likely cause delays to the publication date of your manuscript.

Soon after your final files are uploaded, unless you have opted out, the early version of your manuscript will be published online. The date of the early version will be your article's publication date. The final article will be published to the same URL, and all versions of the paper will be accessible to readers.

For Research, Software, and Methods articles, you will receive an invoice from PLOS for your publication fee after your manuscript has reached the completed accept phase. If you receive an email requesting payment before acceptance or for any other service, this may be a phishing scheme. Learn how to identify phishing emails and protect your accounts at https://explore.plos.org/phishing.

Thank you again for supporting PLOS Computational Biology and open-access publishing. We are looking forward to publishing your work!

With kind regards,

Sharmila Kamatchi

PLOS Computational Biology | Carlyle House, Carlyle Road, Cambridge CB4 3DN | United Kingdom ploscompbiol@plos.org | Phone +44 (0) 1223-442824 | ploscompbiol.org | @PLOSCompBiol
